# Ability of *Brucella melitensis* 16M to Control Nitrogen Process: Identification of a Novel P-II Family Nitrogen Regulator

**DOI:** 10.3390/microorganisms14091898

**Published:** 2026-08-26

**Authors:** Yidan Zhang, Yu Zhang, Shengnan Song, Jing Zhang, Zhihua Sun, Xia Zhou, Chuangfu Chen, Jia Guo, Hui Zhang

**Affiliations:** 1 State International Joint Research Center for Animal Health Breeding, College of Animal Science and Technology, Shihezi University, Shihezi 832003, China; 2 Huyanghe City Haoyuanxin Animal Husbandry Development Co., Ltd., Huyanghe 834034, China; 3 Xinjiang Shawan Tianrun Co., Ltd., Shawan 832100, China; 4 Xinjiang Aheqi Agriculture and Animal Husbandry Development Co., Ltd., Aheqi 843500, China

**Keywords:** Transcriptional regulators, *Brucella melitensis* 16M, FtcR, ChIP-Seq, *gln*B, nitrogen and carbon metabolism

## Abstract

*Brucella* spp. is a bacterium that can survive under conditions of nutrient starvation and is responsible for foodborne illnesses. The OmpR-type transcriptional regulator is characterized by an N-terminal receiver domain and a C-terminal domain, and it plays a regulatory role in diverse physiological processes, notably nitrogen and carbon metabolism. The genome of *Brucella melitensis* 16M contains genes for multiple OmpR-family regulators. However, the genetic program associated with nitrogen metabolism remains elusive. Herein, it was demonstrated that the *B. melitensis* 16M Δ*ftc*R mutant, which lacks the OmpR-type regulator FtcR, displayed compromised viability upon recovery in a nitrogen- and carbon-free medium. Chromatin immunoprecipitation and next-generation sequencing were used to characterize the DNA-binding sites of FtcR. Our genome-wide analysis revealed extensive FtcR-binding sites throughout the *B. melitensis* 16M genome, including the identification of *gln*B as a novel target. As *gln*B encodes a P-II nitrogen regulator, this suggests FtcR plays a direct role in modulating nitrogen, carbon, and energy metabolism under prolonged nutrient starvation. In summary, our findings not only advance our understanding of the transcriptional regulation of nitrogen metabolism but also highlight its critical role in brucellosis pathogenesis, thereby paving the way for the development of novel therapeutics.

## 1. Introduction

The pathogenicity of bacteria has been historically understood as a complex process rooted in molecular determinants, particularly virulence factors that facilitate host niche colonization, replication, and circumvention of immune defenses [1,2]. It follows that successful bacterial replication within a host is contingent upon effective nutrient acquisition and metabolic adaptation to the host microenvironment [3,4]. Virulence is determined not only by pathogen-derived virulence factors but also by the metabolic state and responses of the host [5,6], and this dimension remains among the most underappreciated domains of bacterial pathogenesis.

*Brucella* are typically facultative intracellular pathogens that cause brucellosis [7,8], a serious global zoonosis that produces severe public health concerns and economic losses [9]. Brucellosis is commonly transmitted to animals or humans through direct contact with *Brucella* spp contaminants and through the ingestion of contaminated animal products that are unpasteurized or aerosolized with *Brucella* [10,11,12,13]. The ability to replicate and persist within phagocytic as well as non-antigen-presenting cells is a cornerstone of *Brucella* virulence, where lipopolysaccharide, cyclic β-1,2-D-glucan, phosphatidylcholine, or the type IV secretion system allow it to reside in a membrane-bound compartment known as the *Brucella*-containing vacuole. The inhibition of phagosome-lysosome fusion and intracellular replication within an endoplasmic reticulum-derived compartment are key to this process [14,15]. However, a perspective that is gaining traction suggests these bacteria depend heavily on a “stealth” strategy, which involves minimizing their display of pathogen-associated molecular patterns (PAMPs) [16]. The intravacuolar “diet”-defined by the nutrient milieu encountered during trafficking and within infected organs-is insufficient to permit the synthesis of carbon (C), nitrogen (N), or energy reserves [17]. Metabolic and virulence factors appear to be closely linked, with virulence factors affecting metabolism and vice versa.

A critical determinant of bacterial pathogen virulence is their capacity to adapt central metabolism to exploit the carbon, nitrogen, and energy sources available within the host [18]. Several studies suggest that *Brucella* strains are dependent on glucose catabolism for survival [19,20] and these pathogens utilize gluconeogenic substrates, including glutamate and lactate, to sustain their long-term intracellular residence within mammalian host cells [21,22]. For numerous *Brucella* species, the four-carbon sugar alcohol erythritol constitutes the primary source of both carbon and energy [23]. In the ruminant placenta during late gestation, erythritol is present in substantial quantities and acts as an inducer for multiple *Brucella* genes, such as those involved in T4SS and flagellar assembly [24,25]. The carbon and nitrogen sources for *Brucella* strains can be supplied by glutamate [26], and in the *Brucella*ceae, the precise coordination of NH4^+^ assimilation is mediated by a conserved regulatory machinery comprising the enzymes that interconvert α-ketoglutarate, glutamate, and glutamine, and their associated mirroring mechanisms observed in other bacteria [27]. The significance of this regulatory network for virulence is clearly established. Mutants deficient in its key components exhibit significantly reduced pathogenicity in both cultured mammalian cells and mouse infection models. These critical components include enzymes such as GlnA, GltB, GltD, and GdhZ, as well as regulators like GlnD and GlnE [27,28,29,30,31,32].

In bacteria, two-component systems are among the most common signaling pathways, orchestrating diverse biological processes such as metabolic regulation, stress responses, pathogenesis, resistance to multiple drugs, and interactions with the host [33,34,35,36]. Studies have shown that the OmpR-type regulator regulates various genes related to nitrogen metabolism under the control of a different regulatory protein in bacteria, and function as central regulators governing the expression of major nitrogen metabolism genes (*gln*A, *gln*II, *amt*B, *gln*K, and *gln*D) [37]. Acting as a master regulator, the two-component system navigates the metabolic crossroads of nitrogen and carbon sources to support *Brucella*’s intracellular lifestyle [38,39]. Transcriptomic analysis supports this hypothesis by revealing that two-component system dysfunction impacts genes of carbohydrate and N metabolism [40]. However, little functional information is available regarding the participation of the two-component system, especially the OmpR-type regulator, in N and C metabolism in *Brucella*.

In this study, we demonstrate that the OmpR-type regulator FtcR (BME_RS10945) plays an essential role in enabling *Brucella* to resume growth following nutrient depletion. We used chromatin immunoprecipitation (ChIP) and next-generation sequencing (ChIP-Seq) to characterize the DNA-binding sites of the FtcR protein, including one key FtcR target gene, *gln*B, which is involved in metabolic adaptation to N or C starvation in *B. melitensis* 16M. Our results provide new insights into the regulatory influence of OmpR-type proteins on nitrogen metabolism and virulence pathways in *B. melitensis* 16M.

## 2. Materials and Methods

### 2.1. Bacterial Strains and Cell Line

*B. melitensis* wild-type (wt) strain 16M was cultured in tryptone soya agar (TSA) (Difco, Franklin Lakes, NJ, USA) or tryptone soya broth (TSB) (Difco) under 37 °C and 5% CO_2_. The *B. melitensis* 16M-GFP strain and the *B. melitensis* 16M strain, provided by the Xinjiang Center for Disease Control and Prevention (Urumqi, China), were cultured on TSA supplemented with 30 μg/mL chloramphenicol for plasmid maintenance. All experiments were conducted within a Biosafety Level 3 (BSL-3) laboratory.

### 2.2. B. melitensis 16M Survival Kinetics Under Starvation Conditions

*Brucella melitensis* 16M was grown in TSB at 37 °C under 5% CO_2_ with shaking to an optical density at 600 nm (OD_600_) of 0.4. Phosphate-buffered saline (PBS) was used to wash the bacterial pellet twice, and 10^9^ bacteria/mL were incorporated into two series of triplicate cultures prior to inoculation (50 mL/flask). Bacterial cultures were grown with constant shaking and aeration in a salt solution based on *Brucella* minimal medium, as per established protocols [41]. The solution was free of added carbon and nitrogen sources [42] and was composed of NaCl 128 mM, K_2_HPO_4_ 57 mM, Na_2_S_2_O_3_ × 5 H_2_O 0.4 mM, MgSO_4_ × 7 H_2_O 80 μM, FeSO_4_ × 7 H_2_O 360 nM, MnSO_4_ × H_2_O 600 nM, and CaCl_2_ × 2 H_2_O 272 nM. *B. melitensis* 16M was grown in normal TSB medium as a control. Viability of *B. melitensis* 16M was assessed at the experiment’s initiation and at weekly intervals over five weeks via serial dilution and plating on TSA.

### 2.3. Confocal Laser Scanning Microscopy

A solution lacking sources of carbon or nitrogen was introduced to 24-well plates used for *Brucella* culture. TSB medium was used as the control. In each well, a sterilized clean coverslip (5 × 5 mm, adhesion carrier) was positioned after being autoclaved at 121 °C for 12 min. A 2 mL suspension of *B. melitensis* 16M-GFP (1.0 × 10^9^ CFU/mL) was added to each well on top of the coverslip. The culture plate was incubated at 37 °C in a 5% CO_2_ environment for five weeks, during which the culture medium was refreshed every seven days. Coverslips were gently removed and rinsed three times in PBS, followed by immediate fixation in 2.5% glutaraldehyde for a period of 3 to 5 h at 4 °C. After glutaraldehyde fixation, the samples were washed three times with 1 × PBS, followed by incubation at room temperature for 20 min with a freshly prepared 0.1% sodium borohydride solution. Then, wash three times with 1 × PBS. *Brucella* was examined using a confocal laser scanning microscope (CLSM) ((Nikon C2i+, Tokyo, Japan), and the acquired images were subsequently processed with NIS-Elements Viewer software (version 4.20) (Nikon, Inc., Tokyo, Japan). For each scanning cycle, a three-dimensional volume stack was reconstructed from the two-dimensional fluorescence intensity of all scanned layers. Quantitative analysis of the resulting images was then performed using the Comstat2 program [43].

### 2.4. Bacterial Viability Assay

Bacterial viability was assessed using the LIVE/DEAD BacLight™ kit (Thermo Fisher Scientific, Waltham, MA, USA) [44]. Under a microscope, live cells stain green and dead cells, red. *B. melitensis* 16M was cultured under the conditions described above. A 1 mL aliquot (1.0 × 10^9^ CFU/mL) was passed through a 50 μm filter, pelleted by centrifugation (4000× *g*, 12 min), washed twice with PBS, and then resuspended in 25 μL of Syto9/PI staining solution for a 20 min incubation in the dark. The fluorescent stained cells were observed under CLSM, excite Syto9 with a wavelength of 488 nm and collect green fluorescence; excite PI with a wavelength of 561 nm and collect red fluorescence. And plate counts were used to further verify the viable bacterial counts.

### 2.5. Quantitative Real-Time PCR

For validation of the ChIP-Seq findings, the ten most enriched target genes were subjected to quantitative real-time PCR (qRT-PCR) analysis. The wild-type (wt) strain and its derivatives were pelleted by centrifugation (8500× *g*, 15 min). Total RNA was isolated from the pellet using TRIzol (TransGen Inc., Beijing, China). The RT reagent kit (TransGen Inc., China) was utilized for the reverse transcription of RNA, while cDNA amplification was carried out in a 20 µL reaction mixture that included 2× SYBR Premix Ex Taq II (Takara Inc., Kusatsu, Japan). The primers utilized are detailed in Table 1. qRT-PCR was performed using a QuantStudio™ 7 Flex system (Thermo Fisher Scientific, Waltham, MA, USA). The thermal cycling protocol included an initial pre-incubation at 95 °C for 5 min, followed by 40 amplification cycles of 95 °C for 35 s, 56 °C for 35 s, and 72 °C for 40 s. All experiments were performed in triplicate.

### 2.6. Construction of Mutant and Complemented Strains

**Figure A1:**
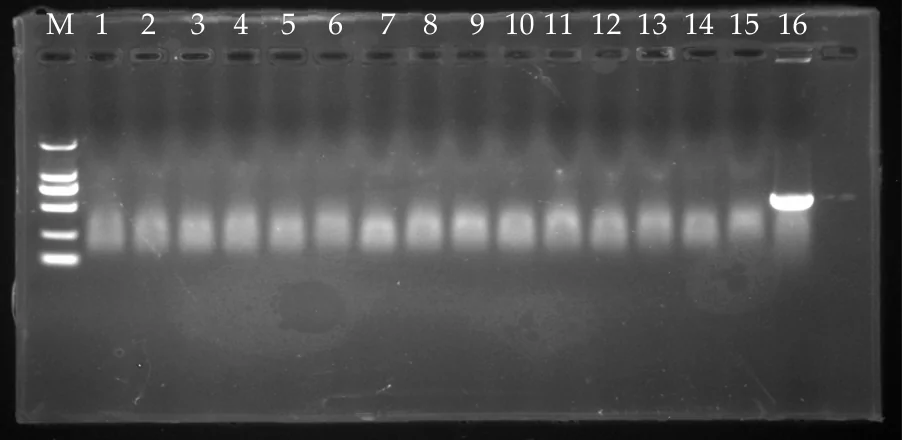
*ftc*R deletion mutant validation. M: DNA marker; 1**–**15: sample; 16: positive control.

**Figure A2:**
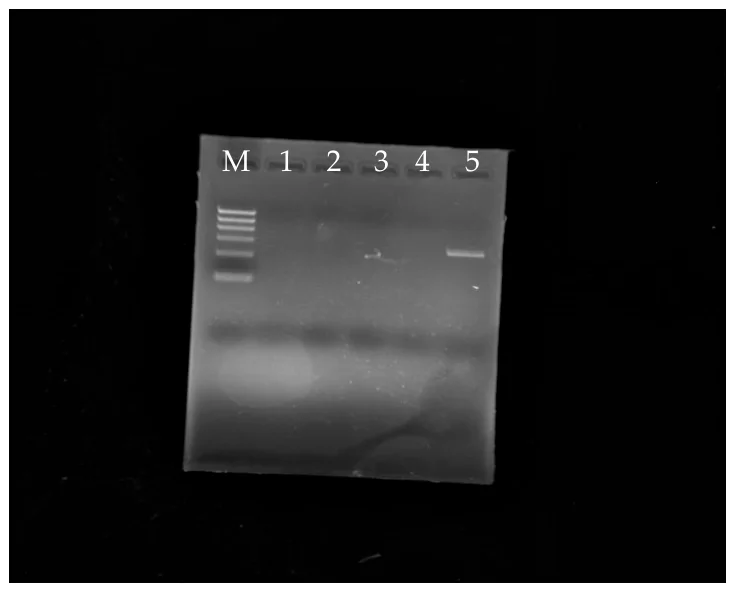
*gln*B deletion mutant validation. M: DNA marker; 1**–**4: sample; 5: positive control.

The deletion mutants in *ftc*R and *gln*B were generated using previously published methods [45]. An overview of the primers used to construct the mutants is shown in Table 2. In brief, KpnI restriction enzyme sites were added to the 5’ end of Kan-F, and PstI restriction enzyme sites were added to the 5’ end of Kan-R. Kan-F and Kan-R primers were used to amplify the kanamycin gene from pBBR1MCS-4. PUC19K was generated by subcloning PCR products into the pMD19-T Simple Vector (TransGen Inc.) via the KpnI and PstI sites and sequencing the resulting PCR products. A suicide plasmid was generated by amplifying the upstream and downstream homologous fragments of the target gene. The pUC19K-ftcR and glnB plasmids were introduced into electrocompetent *B. melitensis* 16M cells by electroporation. Successfully constructed FtcR- and glnB-deficient strains (ΔftcR, ΔglnB). Transformants were selected using agar plates supplemented with ampicillin (50 µg/mL) and kanamycin (50 µg/mL). To complement the mutants, we used pBBR1MCS-4 plasmids containing the native genes [46]. We successfully constructed the complementation strains ftcR and glnB (ΔftcR-C, ΔglnB-C). Results are shown in Figure A1 and Figure A2 in the Appendix A.

### 2.7. Measurement of β-Galactosidase Activity

The reporter fusion pBBCmpftcR-lacZ, consisting of pftcR-lacZ, was created following the previously outlined methods [47,48,49]. β-Galactosidase assays were performed following the Miller protocol as described by Fretin et al. [47].

### 2.8. Western Blot

**Figure A3:**
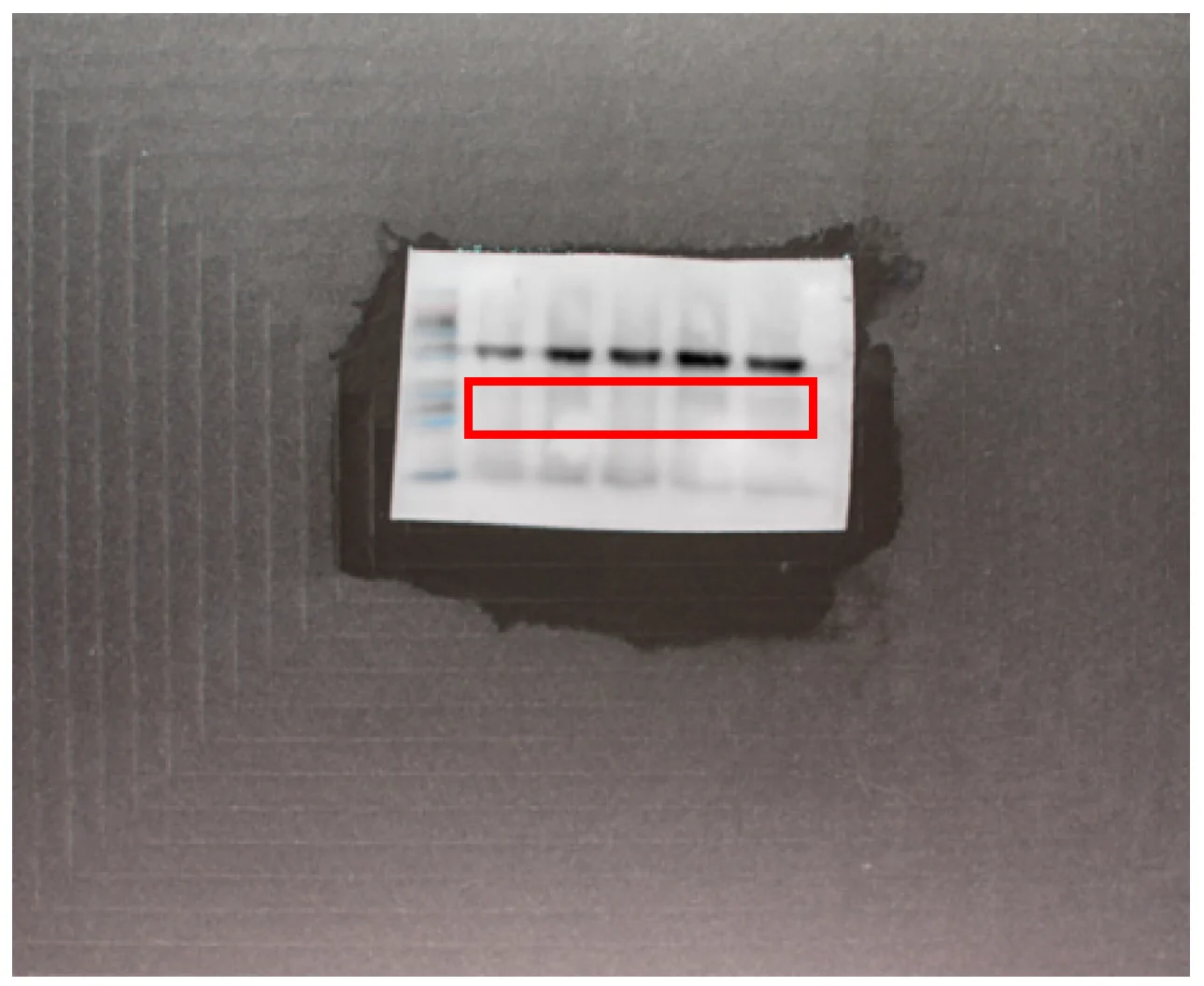
FtcR WB verification results.

**Figure A4:**
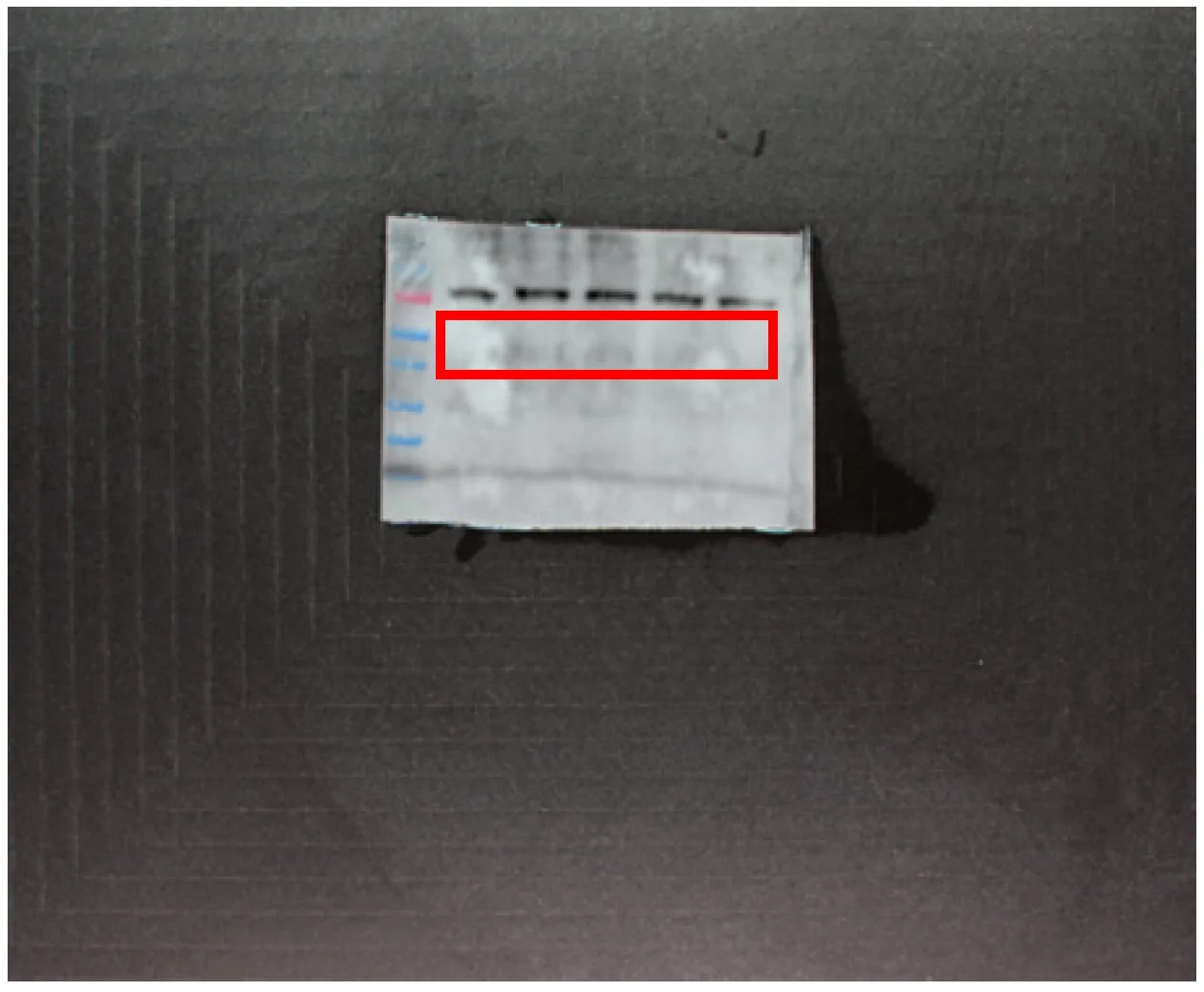
FliF WB verification results.

**Figure A5:**
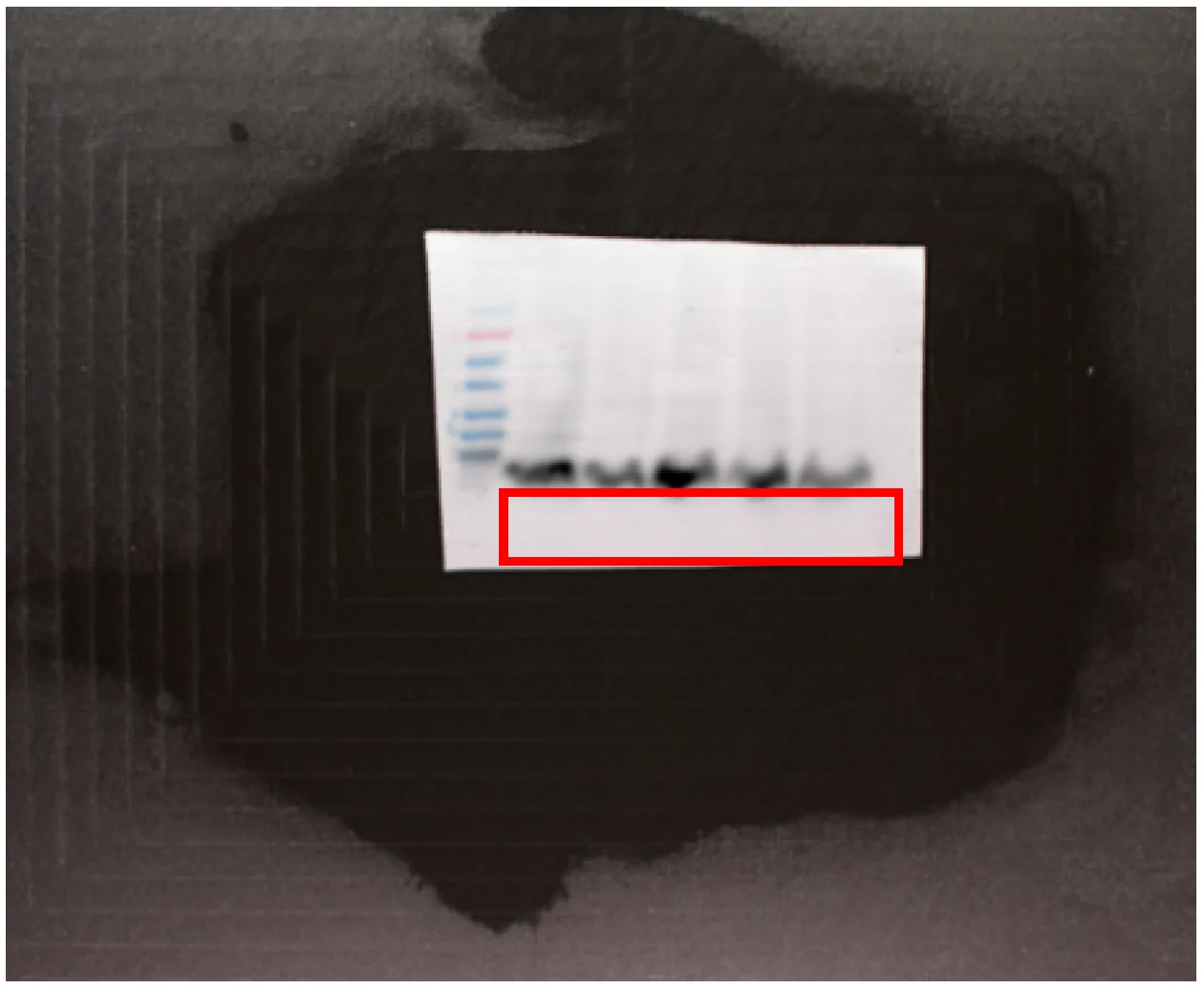
Omp10 WB verification results.

The bacterial pellets were washed twice with PBS and subsequently treated with DNase I to reduce nucleic acid contamination, followed by the addition of protease inhibitors. Following centrifugation at 16,000 × g for 30 min at 4 °C, the supernatant was collected and its protein concentration was quantified using the BCA protein assay kit (Thermo Fisher Scientific, Waltham, MA, USA). Samples were combined with SDS-PAGE loading buffer, heated at 100 °C in a water bath for 10 min, and subsequently resolved on a 12% polyacrylamide gel. A monoclonal anti-Omp10 antibody was used as the loading control [50]. The gel was subsequently transferred to a polyvinylidene fluoride membrane and incubated with 5% skim milk powder at 37 °C for 2 h for blocking treatment. After three washes with TBST, FtcR- and FliF-specific antisera (diluted 1:1000) were added and incubated overnight at 4 °C. The membrane was washed three times with TBST (10 min per wash). It was then incubated with peroxidase-conjugated anti-rabbit IgG antibody (diluted 1:5000) for 1 h at 37 °C with shaking. Bands were detected using Protein Simple (FluorChem E, Costa Mesa, CA, USA). Relative protein expression levels were analyzed and graphically represented using Image J 1.8.0_345 software (NIH, Bethesda, MD, USA). Results are shown in Figure A3, Figure A4 and Figure A5 in the Appendix A.

### 2.9. Chromatin Immunoprecipitation and High-Throughput Sequencing

ChIP assays were performed as described previously [48]. The raw sequencing reads from the ChIP-seq experiments have been archived in the NCBI database with the accession number PRJNA871752.

### 2.10. Protein Purification and Electrophoretic Mobility Shift Assays (EMSAs)

The resulting products of FtcR protein have been described in our previous work [48]. Biotin labeling of the DNA probe was achieved via a two-step PCR procedure. Specifically, a 200 bp amplicon encompassing the target motif was generated using the EMSA F/R primers. To generate the biotin-labeled probe DNA, a biotin-labeled probe primer was used to amplify the product. Probes for *gln*B deletion were constructed by means of overlapping PCR. Transfer the reaction mixture containing 30,000 cpm of probes and gradient-concentrated ftcR protein (0–200 ng) into a gel electrophoresis buffer system comprising 1 µL dithiothreitol (20 mM), 1 µL bovine serum albumin (10 mg/mL), and 4 µL 5 x binding buffer (100 mM Tris-HCl, pH 7.5; 5 mM magnesium chloride; 0.5 mM EDTA; 50% glycerol), adjusting the final reaction volume to 20 µL; incubate at room temperature for 35 min. The samples were analyzed using 6% PAGE. DNA in the gels was visualized by staining with nucleic acid dye for 10 min, and images were captured using a gel documentation system (Bio-Rad, Hercules, CA, USA).

### 2.11. Statistical Analysis

Statistical analyses were conducted with the aid of GraphPad Prism 9.0 software (GraphPad Inc., La Jolla, CA, USA). The results are shown as mean ± standard deviation. The data passed the Shapiro–Wilk test for normality and the Levene’s test for homogeneity of variances. Statistical analyses were performed using an unpaired, one-way or two-way analysis of variance (ANOVA). Significant interactions were further dissected using simple effects analysis, with pairwise comparisons adjusted by Tukey’s post hoc test. Probability (*p*) values were deemed statistically significant when *p* < 0.05. Each experiment shall undergo three biological replicates; the results from these three experimental repetitions shall be used for calculations.

## 3. Results

### 3.1. Characterization of the Transcription of OmpR-Like Regulators Under Extreme Starvation Conditions

**Figure 1:**
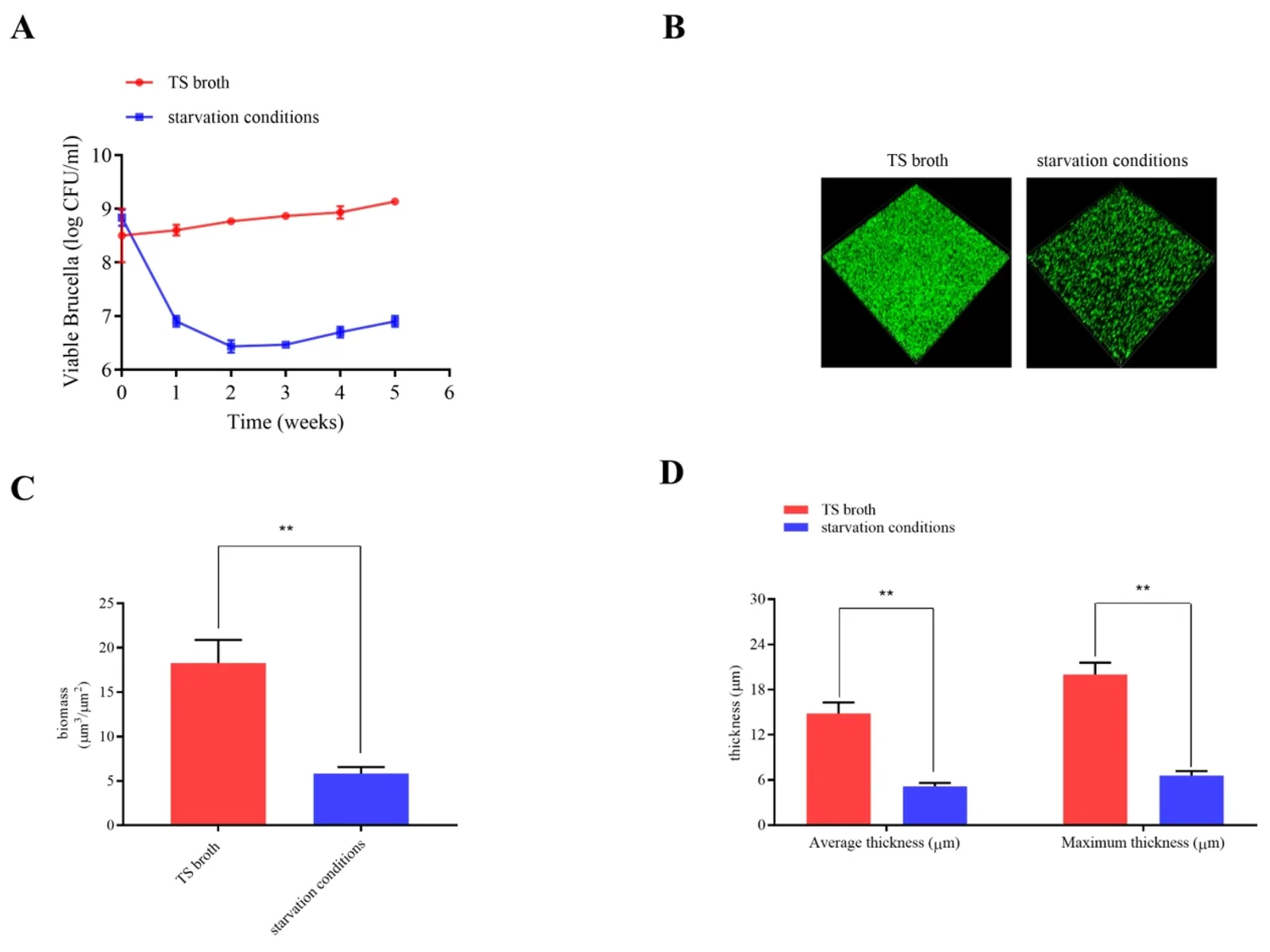
Analysis of the survival kinetics of *B. melitensis* 16M under starvation conditions and control group. (**A**) Survival kinetics of *B. melitensis* 16M in starvation conditions (blue curve) or TS broth medium (red curve) over a period of five weeks at 37 °C. (**B**) Live confocal imaging of stacks of *B. melitensis* 16M-GFP grown for five weeks in TS broth or salt solution. Scale bars = 20 µm. (**C**,**D**) Confocal images were subjected to quantitative analysis using the Comstat2 program to determine the biomass (**C**) and the average and maximum thickness (**D**) in starvation conditions and control group. All results shown are derived from three independent biological replicate experiments. The data are shown as average ± standard deviation; statistical analysis was performed using one-way ANOVA. ** *p* ≤ 0.01.

Based on early studies on *M. tuberculosis* and *Brucella suis*, we established a model medium with no C and N sources to study the effect of starvation on the long-term viability of *B. melitensis* 16M. A steady increase in the number of bacterial cells was observed over five weeks in rich medium, whereas a sharp decline of approximately 2.0 logs in the number of viable bacteria was observed over one week under starvation conditions (Figure 1A). Parallels have been postulated between the stationary-phase physiology and the physiological state of *Brucella* in the intracellular niche. After five weeks of stationary-phase culture, CLSM results showed that *B. melitensis* 16M exhibited a defective phenotype under starvation conditions, as evidenced by the bacterial biomass, which was 5.84 μm^3^/μm^2^ on average (Figure 1B,C); and average and maximum aggregates thickness were recorded as 5.16 μm and 6.8 μm, respectively (Figure 1B,D). The bacterial biomass under starvation conditions was four-fold less than under control conditions. We performed viability tests with the LIVE/DEAD bacterial viability kit and confirmed that these cells maintained their capacity to grow in rich medium and starvation conditions, showing that more live bacteria were detected in the rich medium (Figure S1A,B); however, *B. melitensis* 16M also survived under extreme starvation conditions (Figure 1 and Figure S1).

**Figure 2:**
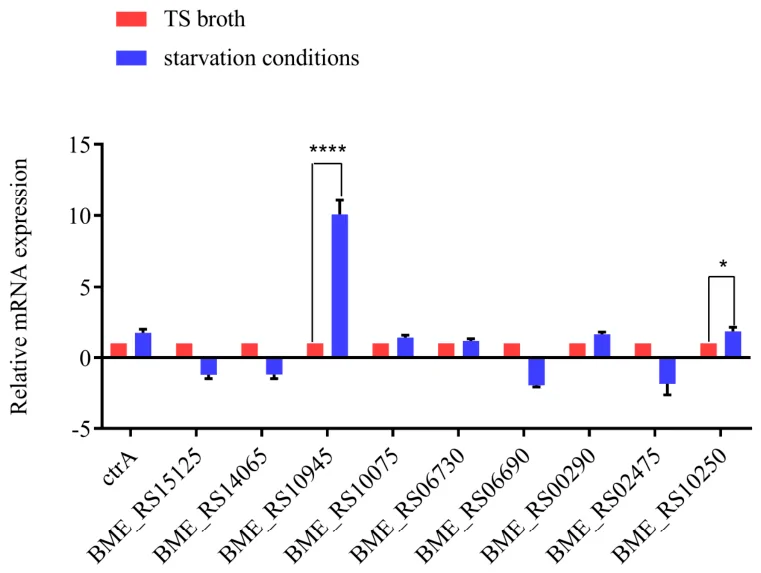
10 OmpR-type regulators were evaluated by quantitative reverse transcription PCR (qRT-PCR) assays between the *B. melitensis* 16M in starvation conditions and control group. The 10 OmpR-type regulator’s expression levels were further detected by qRT-PCR. The results at each time point are expressed as the means ± standard deviations from three independent biological replicate experiments. Statistical analysis was performed using one-way ANOVA; * *p* < 0.05, **** *p* < 0.0001.

Considering that OmpR-like regulators are involved in the transcriptional control of important processes of the N or C metabolism, we first profiled the transcriptional expression of ten OmpR-type regulators annotated in the National Center for Biotechnology Information (NCBI) database (Table 3) that have not been characterized in the N or C metabolism. The results showed that the transcription levels of BME_RS15125, BME_RS14065, BME_RS06690, and BME_RS02475 were reduced under starvation conditions (Figure 2), compared with those of the rich medium. The transcript levels of *ctr*A, *ftc*R, BME_RS10075, BME_RS06730, BME_RS00290, and BME_RS10250 increased (Figure 2). Notably, the *ftc*R gene was significantly elevated under starvation conditions (Figure 2). The *ftc*R-encoded FtcR-like protein is involved in virulence during the *B. melitensis* 16M life cycle.

### 3.2. FtcR Is Required for Recovery from Starvation

**Figure 3:**
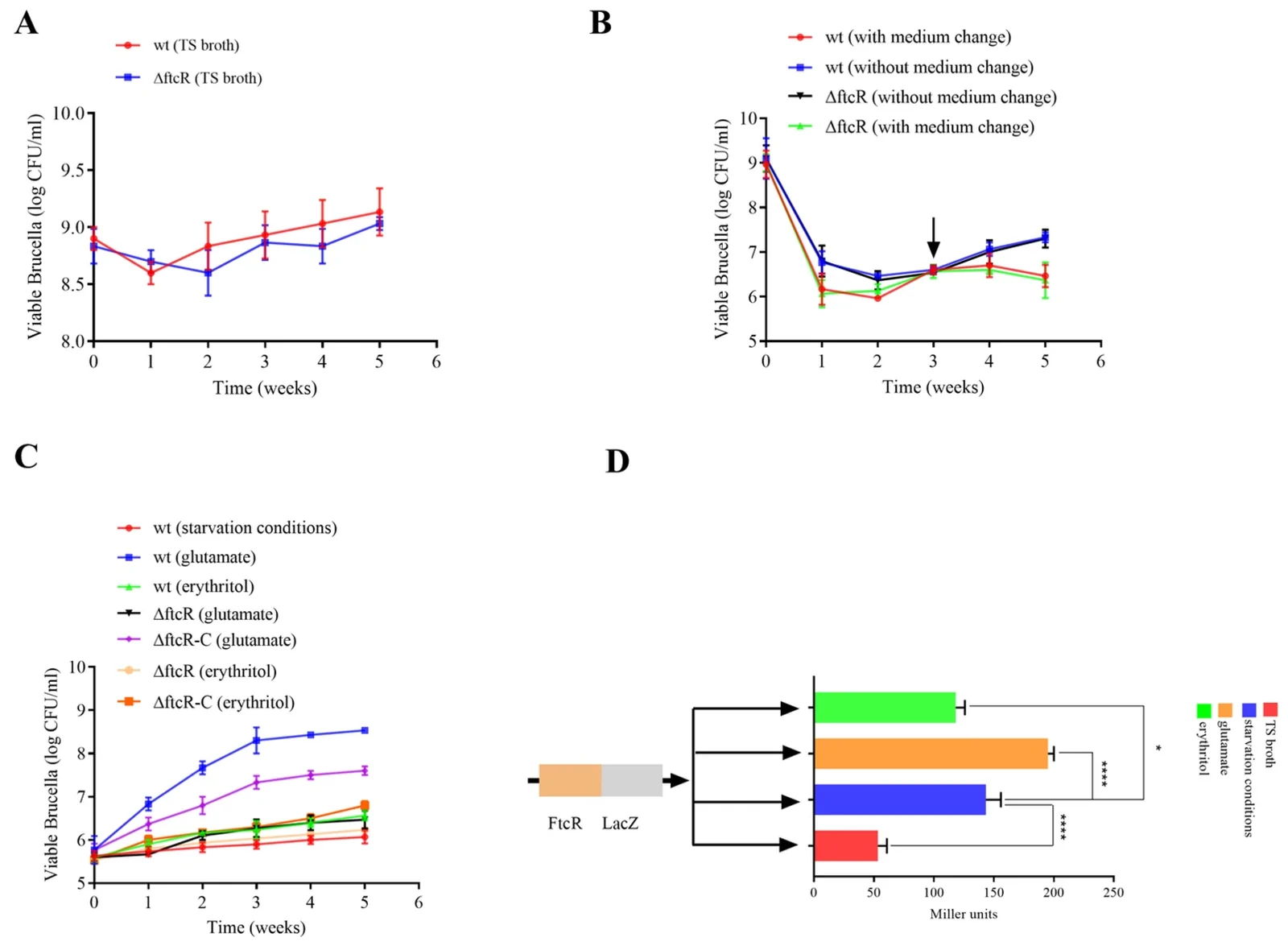
Functional analysis of Δ*ftc*R in response to nitrogen or carbon changing. (**A**) Survival kinetics of wt and Δ*ftc*R strains in TS broth medium over a period of five weeks at 37 °C. (**B**) Survival kinetics of Δ*ftc*R and wt strains in a salt solution over a period of five weeks at 37 °C with or without medium change. (**C**) Analysis of the recovery ability of wt, Δ*ftc*R and Δ*ftc*R-C strains from nitrogen or carbon starvation after three weeks of incubation under starvation conditions. (**D**) The effect of different conditions on expression of the activity of the *ftc*R promoter region was detected by β-galactosidase assays using an *ftc*R promoter-lacZ transcriptional fusion reporter plasmid. The data are shown as average ± standard deviation; statistical analysis was performed using two-way ANOVA to assess main effects and interactions; *p* < 0.05 was considered the threshold for statistical significance; * *p* < 0.05, **** *p* < 0.0001. Black arrow: The survival dynamics of the Δ*ftc*R strain and the wt strain overlap when continuously cultured in saline (with or without changing the medium) for three weeks.

A steady increase in the number of wt and Δ*ftc*R cells was observed over five weeks in rich medium. Although the number of FtcR cells was slightly reduced after one week compared to that of the wt, it was not statistically significant (Figure 3A). Exposure of both wild-type (wt) and Δ*ftc*R cells to a carbon- and nitrogen-depleted salt solution triggered a sharp drop in viability, amounting to an approximately 3.0-log reduction within two weeks (Figure 3B), followed by stabilization of the number of viable bacteria during the following three weeks (Figure 3B). This behavior indicated adaptation of a subpopulation of the pathogen to the environmental conditions encountered. The *B. melitensis* 16M growth curves under nutrient starvation were similar to those of *Mycobacterium* spp. A crucial consideration is the long-term survival of a starvation-resistant subpopulation and the dynamic interplay between bacterial death and the replication sustained by nutrients released from lysed *Brucella* cells. Washing the bacteria and medium replacement following three weeks of incubation did not affect the bacterial survival kinetics (Figure 3B, red curve), indicating that soluble metabolites derived from lysed bacteria may have a limited contribution. Furthermore, no marked difference in growth was detected between the wild-type and *ftc*R mutant under starvation conditions.

To explore the role of FtcR in C and N metabolism in vitro, we used glutamate and erythritol as the sole N and C sources after the starvation phase. Glutamate and erythritol can function as N, C, and energy sources when supplied alone to the *Brucella* basal medium. As shown in Figure 3C, wt cells grew rapidly with the addition of glutamate and erythritol from approximately 5.5 log CFU/mL to approximately 8.5 and 5.5 log CFU/mL, respectively (Figure 3C), whereas the cells grew very slowly under starvation conditions. Our findings align with previous research, showing that glutamate and erythritol can be used as the sole C or N sources for *Brucella* in vitro. However, the Δ*ftc*R strain did not recover from starvation to normal growth with the addition of glutamate or erythritol (Figure 3C), and the Δ*ftc*R-C strain reverted to this phenotypic change in glutamate (Figure 3C). No statistical differences between wt and Δ*ftc*R strains were observed when adding erythritol (Figure 3C). In addition, the glutamate added to the wt alleviated the inhibitory effects of starvation in *B. melitensis* 16M; however, recovery from the N starvation phenotype was disrupted in Δ*ftc*R treated with glutamate after starvation, and average bacteria biomass was 3.66 μm^3^/μm^2^ (Figure S2A,B), average and maximum thickness were 4.06 and 4.97 μm, respectively (Figure S2A,C), and cells presented two-fold less biomass than that of the wt.

A transcriptional reporter assay utilizing an ftcR-lacZ fusion plasmid was performed to evaluate the role of FtcR in metabolic regulation. The ftcR-lacZ plasmid was constructed and transformed into wt in TS broth under starvation conditions, and with the addition of glutamate and erythritol. Compared to that in the TS broth, beta-galactosidase activity increased under starvation conditions and was significantly different with the addition of glutamate (Figure 3D). Collectively, our results suggest that FtcR is involved in the *B. melitensis* 16M N metabolism during starvation and permits rapid growth after N starvation when FtcR is present, especially when glutamate is the sole C source.

### 3.3. FtcR Binds to a Large Number of Sites Across the B. melitensis 16M Genome

**Figure 4:**
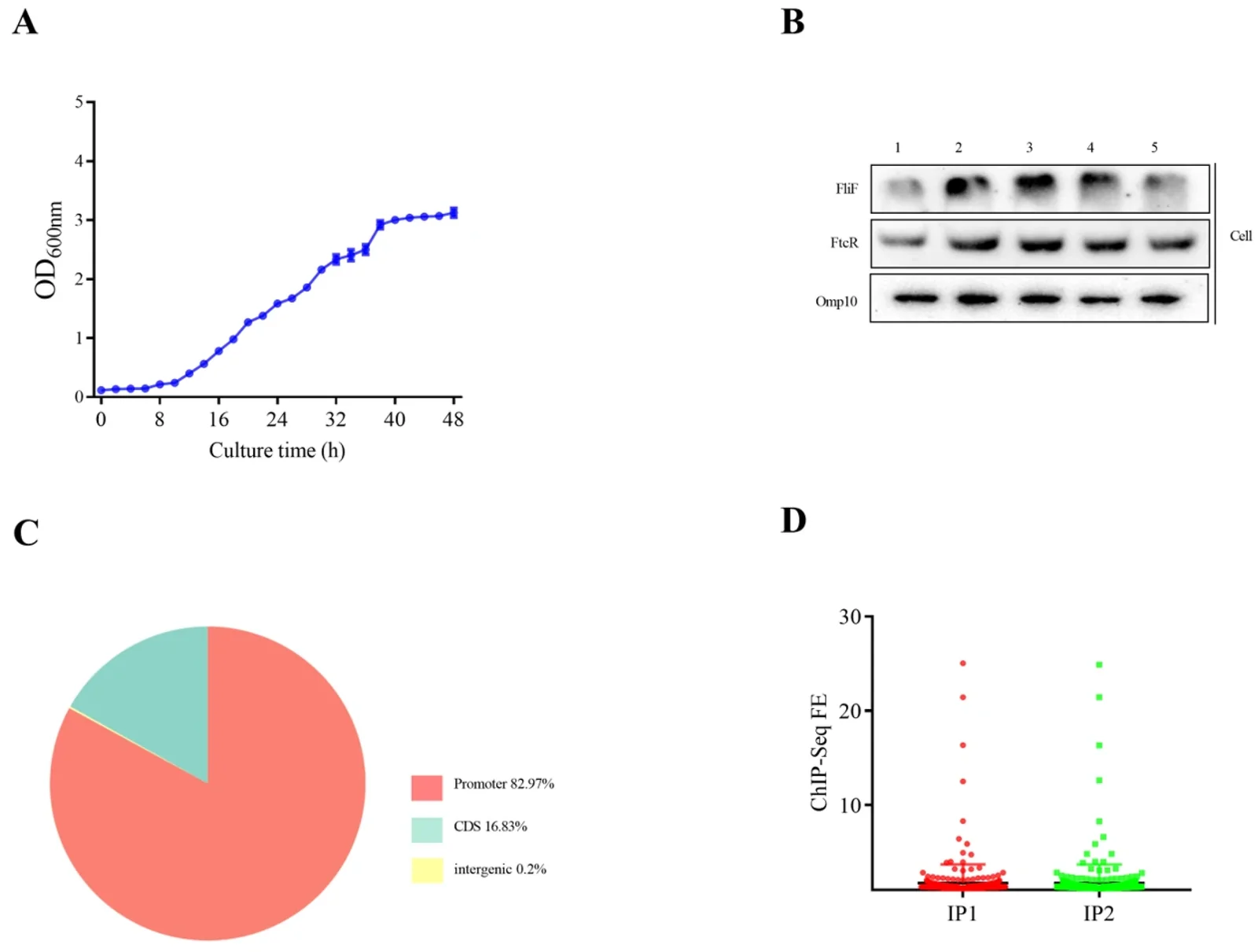
ChIP-Seq analysis of the binding sites of FtcR across the *B. melitensis* 16M genome. (**A**) The 48 h growth curve of *B. melitensis* 16M. (**B**) The strains were cultivated in TS broth and extracts were prepared from samples harvested at the end of the latent phase of growth (OD600 nm= 0.1 and 0.2; lanes 1 and 2), the beginning of the exponential phase (OD600 nm= 0.3 and 0.4; lanes 3 and 4), and the exponential phase (OD600 = 0.5; lanes 5). The expression of FtcR and one of its direct targets FliF was detected by Western blotting. (**C**) Peak locations across the genome. (**D**) Scatter plot showing distribution of the observed ChIP-seq FE values.

We speculated that FtcR might be involved in N metabolism in *B. melitensis* 16M. Based on our previous study, we performed the FtcR regulon through ChIP and high-throughput sequencing. More specifically, we used mid-exponential bacteria as the starting material, in which the target protein FliF did not reach its peak expression. Western blot analysis showed a clear increase in FliF production at the beginning of the exponential phase (OD_600 nm_ 0.3 and 0.4; lanes 3 and 4) (Figure 4A,B). The expression of FtcR and the target proteins reached a peak in the wt strain during the exponential growth phase (OD_600 nm_ 0.3 to 0.4) (Figure 4A,B), suggesting the de novo synthesis of this regulator and its direct target gene. Thus, we chose the stage of de novo expression of FtcR at an OD_600 nm_ 0.3 and then performed ChIP using a polyclonal antiserum against the native FtcR protein. Genome-wide next-generation sequencing was performed to analyze the immunoprecipitated FtcR DNA fragments. ChIP-Seq peaks (363) were located in the promoter, CDS, and intergenic regions across the genome (Figure 4C). Analysis of the binding distribution for all identified ChIP-Seq peak summits (Figure S3A), calculated as the distance to the nearest gene in the *B. melitensis* 16M genome, showed a preference for regions about 200 bp upstream of start codons (Figure S3B). Lastly, a broad dynamic range was observed in the FE intensity values from the ChIP-Seq data (Figure 4D); potential explanations for this include heterogeneity in FtcR-binding affinity at individual sites, variation in the number of bound FtcR molecules, or steric hindrance from other DNA-binding proteins.

### 3.4. Characterization of FtcR-Binding Sites Through GO and KEGG Analyses

**Figure 5:**
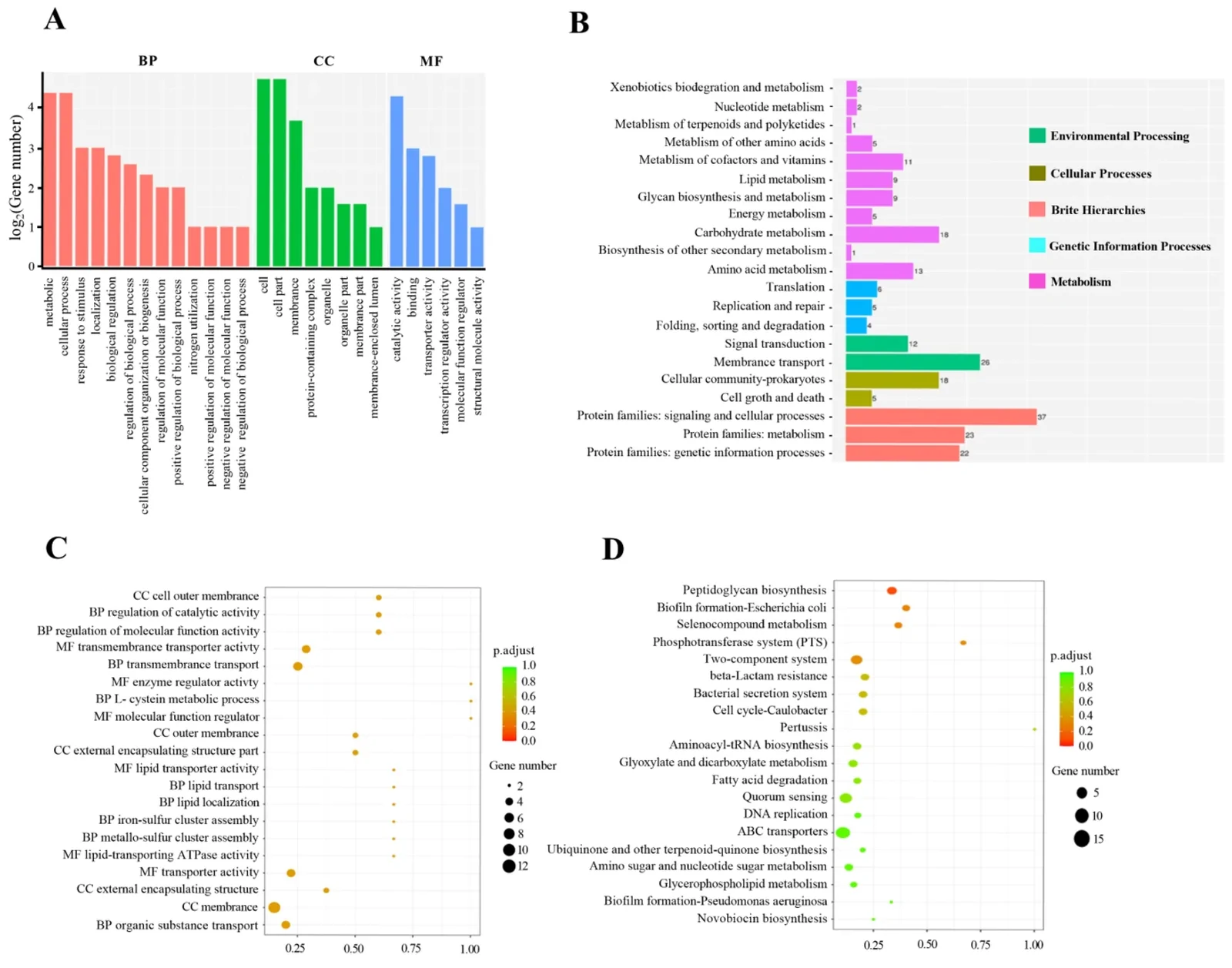
GO and KEGG analyses of the binding sites of FtcR. (**A**) GO terms analysis of the number and function of enriched genes of the binding sites of FtcR. BP, biological process; CC, cellular component; MF, molecular function. (**B**) KEGG clustering and classification of enriched genes of the binding sites of FtcR. (**C**,**D**) GO and KEGG analyses of the enriched genes of the binding sites of FtcR. The rich factor represents the ratio of enriched gene numbers annotated in this pathway term to all gene numbers annotated with this pathway term. A greater rich factor indicates a greater degree of pathway enrichment. The padj represents the corrected *p*-value and ranges from 0 to 1, and a lower value indicates greater pathway enrichment.

To gain functional insights into the identified FtcR target genes, we categorized them and performed pathway analysis using the GO and KEGG databases. We assigned these genes to the three major GO categories—cellular components, molecular functions, and biological processes—leveraging the Gene Ontology database. Within the biological process ontology, metabolic, cellular, and response to stimulus processes were the most significantly enriched (Figure 5A). Within the cellular component ontology, the most significantly enriched terms were “cell” and “cell part” (Figure 5A). Within the molecular function ontology, catalytic activity, binding, and transporter activity were the most predominantly represented (Figure 5A). In addition, the products encoded by FtcR-binding genes were classified into five main categories (environmental processing, cellular processes, brite hierarchies, genetic information processes, and metabolism) using the KEGG database. The top two metabolic pathways were cofactor, vitamin, and carbohydrate metabolisms (Figure 5B). Translation, replication and repair, and folding, sorting, and degradation are involved in genetic information processes (Figure 5B). The two enriched terms in the environmental processing category were signal transduction and membrane transport (Figure 5A). The most abundant subcategories in the brite hierarchy were protein families, signaling, and cellular processes (Figure 5B). Cellular community prokaryotes, cell growth, and cell death were involved in these cellular processes (Figure 5B).

To characterize the signaling pathways involved in these genes, 40 pathways were significantly enriched in GO and KEGG pathway analyses, and we selected the top 10 enriched pathways based on *p.adjust* to construct a network map of the GO and KEGG signaling pathways. Through GO analysis, BME_RS14320, BME_RS02005, and BME_RS00635 are involved in the outer membrane and external encapsulation structure (Figure S4). BME_RS10250, *glnB*, and *nrdH* are involved in the regulation of MF molecular function, MF enzyme regulation, BP regulation of molecular function, and BP regulation of catalytic activity (Figure S4). BME_RS09580, BME_RS00255, *mscL*, BME_RS04905, BME_RS01260, BME_RS02195, and BME_RS02355 are involved in MF and BP transmembrane transport (Figure S4). BME_RS10050 and BME_RS06105 are involved in the BP L-cysteine metabolic process (Figure S4). In KEGG analysis, *pstP* and BME_RS10060 are involved in the phosphotransferase system. BME_RS05315, BME_RS14185, *pyC*, *sdh*C, and BME_RS10010 are involved in C fixation pathways in prokaryotes (Figure S5). BME_RS04935, BME_RS04065, BME_RS02895, BME_RS06800, BME_RS05275, BME_RS09065, *mur*G, *bep*D, *clp*X, *dna*A, BME_RS02120, BME_RS06670, bepC, BME_RS04335, *ntr*Y, *rpo*N, *gln*B, BME_RS10070, BME_RS02470, BME_RS02475, BME_RS14065, BME_RS08725, *dks*A, BME_RS10340, *sec*E, *sec*DF, and BME_RS10315 are involved in peptidoglycan biosynthesis, vancomycin resistance, beta-lactam resistance, cell cycle-caulobacter, two-component system, pertussis, bacterial secretion system, and biofilm formation (Figure S5).

### 3.5. FtcR Interacts with the glnB Promoter in Vitro

**Figure 6:**
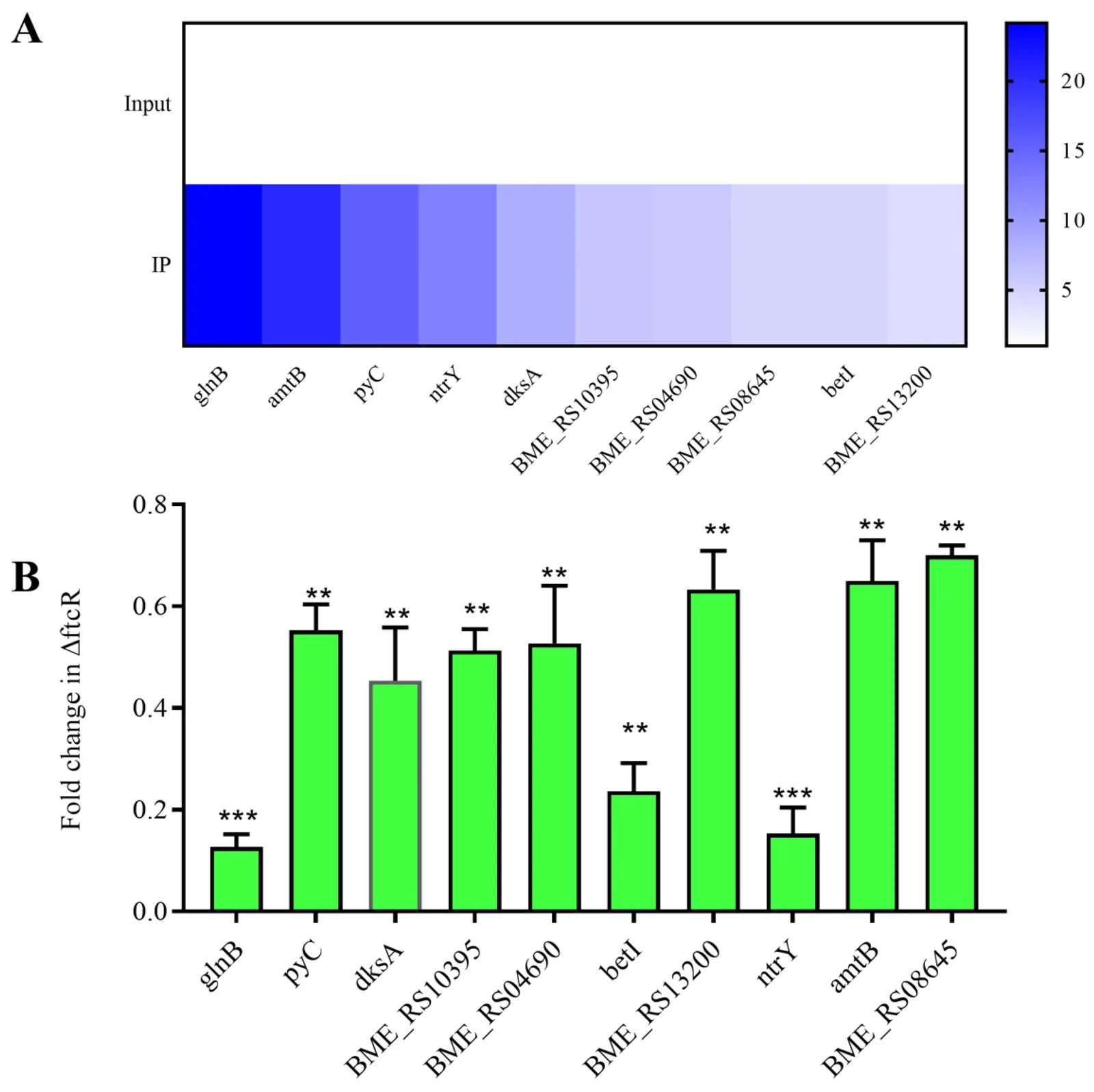
The enriched genes of the binding sites of FtcR were evaluated by quantitative reverse transcription PCR (qRT-PCR) assays. (**A**) The heatmap shows the expression levels of the top 10 enrichment target genes between the Input and IP. (**B**) The top 10 enrichment target gene expression levels were further detected by qRT-PCR. The results at each time point are expressed as the means ± standard deviations from three independent biological replicate experiments. Statistical analysis was performed using two-way ANOVA; ** *p* < 0.01, *** *p* < 0.001.

**Figure 7:**
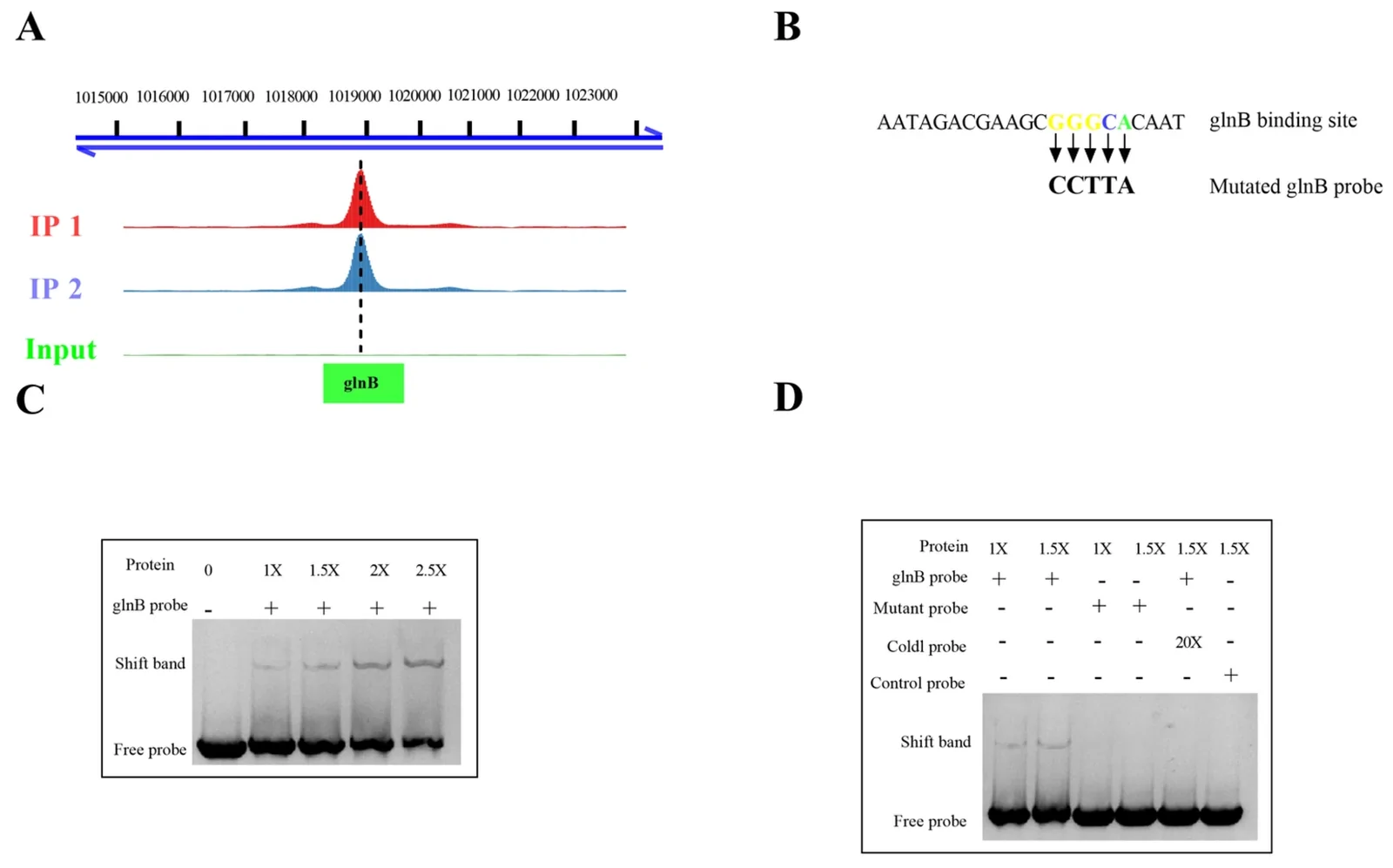
The FtcR binds to the promoter region of *gln*B in vivo and in vitro. (**A**) FtcR-enriched sequence reads located within the promoter regions of *gln*B, with annotated genes shown at the bottom, ChIP1 and ChIP2, from two independent experimental replicates. Scale bar: 1 kb. (**B**) The conserved binding motif binding sites of FtcR. (**C**) EMSA for FtcR binding to the GGGCA motif in the *gln*B promoter’s fragments, which was labeled by biotin. (**D**) Specificity and competition assays. The GGGCA motif was mutated into CCTTA to test for sequence specificity. Unlabeled cold *gln*B promoter region was tested for competition with biotin-labeled *gln*B promoter. Unlabeled cold *gln*B promoter region inhibits the binding of FtcR to the labeled promoter. The symbols “–“ and “+” indicate the absence and presence of the corresponding proteins or probes. Unrelated probes were used as the negative controls. Results are representative of at least three independent experiments.

To ascertain the FtcR dependence of target gene regulation, we analyzed the expression of top ten representative enriched genes via quantitative PCR (qPCR) in both wild-type and ftcR mutant strains. Relative to the wild-type, the ftcR mutant showed a significant (*p* < 0.01) decrease in the expression of most target genes (Figure 6A,B). ChIP-Seq analysis identified the *gln*B promoter region as the most significantly enriched target (Figure 7A). To verify whether FtcR could bind to the promoter regions of these putative target genes and play a regulatory role, EMSAs were performed using fragments of these sequences and the FtcR protein. The ChIP-seq data enabled us to refine the consensus binding motif for FtcR. Using the HOMER tool to analyze the identified peaks, we identified a 5 bp FtcR consensus sequence (5’-GGGCA-3’) (Figure 7B). The in silico prediction of FtcR-binding sites in the *gln*B promoter was substantiated by EMSA, which verified the specific interaction between FtcR and the motif-containing promoter region in vitro (Figure 7C). The interaction was abolished by either excess unlabeled competitor DNA or by introducing mutations into the motif, thereby demonstrating the specificity of the FtcR-glnB promoter binding (Figure 7D).

### 3.6. GlnB Was Responsible for the Response to Recovery from N Starvation

**Figure 8:**
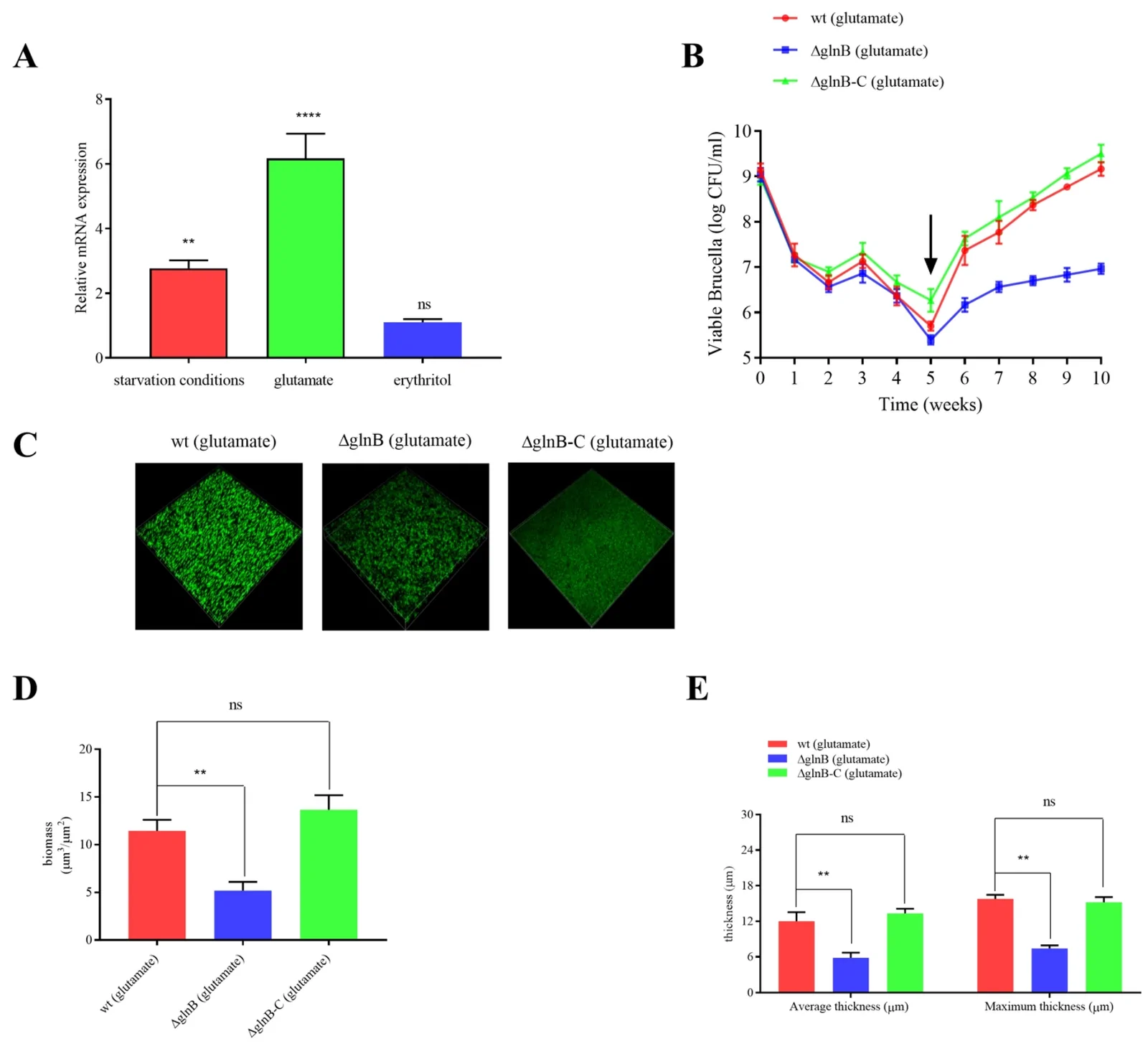
Functional analysis of the *gln*B in adapting to nitrogen starvation. (**A**) The *gln*B expression levels were detected by qRT-PCR under different conditions. The results at each time point are expressed as the means ± standard deviations from three independent biological replicate experiments. (**B**) Analysis of the recovery ability of wt, Δ*gln*B and Δ*gln*B-C strains from nitrogen or carbon starvation after five weeks of incubation under starvation conditions. Black arrow: The wt, ΔglnB, and ΔglnB-C strains overlap in their ability to recover from nitrogen or carbon starvation during the fifth week of cultivation under starvation conditions. (**C**) Live confocal imaging of stacks of *B. melitensis* 16M-GFP grown for five weeks in adding glutamate conditions. Scale bars = 20 µm. (**D**,**E**) Confocal images were subjected to quantitative analysis using the Comstat2 program to determine the biomass (**D**) and the average and maximum thickness (**E**) in adding glutamate conditions. All results shown are derived from three independent biological replicate experiments. The data are shown as average ± standard deviation; statistical analysis was performed using two-way ANOVA; *ns* *p* > 0.05, ** *p* < 0.01, **** *p* < 0.0001.

Previous studies demonstrated the role of GlnB in the regulation of N metabolism. FtcR occupancy sites were found upstream of *gln*B, suggesting the direct regulation of these factors by FtcR. Here, we examined *gln*B transcription levels under different N conditions. Compared to the rich medium, the transcription level of *gln*B increased (Figure 8A). Moreover, the transcript level of *gln*B was significantly elevated when glutamate was the sole N or C source (Figure 8A). When only erythritol was added, the *gln*B transcription level did not change (Figure 8A).

**Figure 9:**
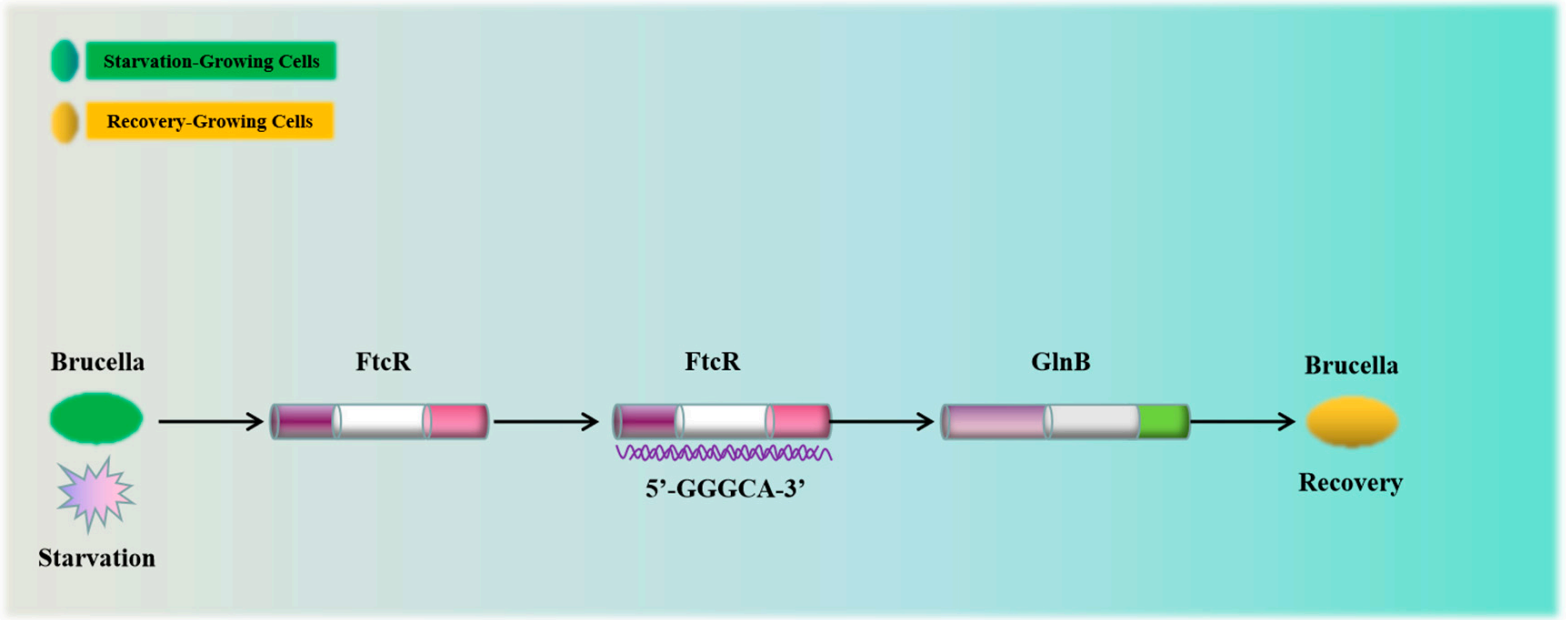
Regulatory mechanism of the transcriptional factor FtcR to control nitrogen process. Under starvation conditions, the transcriptional factor FtcR plays an essential role in controlling recovery from starvation by directly binding to the conserved GGGCA motif upstream of *gln*B, which is involved in regulating *B. melitensis* 16M nitrogen process when challenged by starvation.

Significant sequence homology was identified between GlnB and members of the P-II protein family through analysis against the Conserved Domain Database (CDD) (Figure S6A,B). Functional prediction analysis indicated ‘nitrogen regulator’ as the most significant term. Furthermore, protein–protein interaction (PPI) networks offer a more comprehensive view of functional linkages between proteins (Figure S6A). PPI network analysis was utilized to systematically predict and evaluate the primary known and potential protein interactors of *gln*B (Figure S6C). We thus investigated whether *gln*B affected *B. melitensis* 16M growth under starvation conditions via comparing the survival of wt, Δ*gln*B, and Δ*gln*B-C in starvation conditions and adding glutamate as the sole N or C source. The wt, Δ*gln*B, and Δ*gln*B-*C* strains were first activated in rich medium and then transferred into the medium with different growth conditions. After incubation, the CFUs were determined weekly. As shown in Figure 8B, the wt, Δ*gln*B, and Δ*gln*B-*C* strains are not statistically different during the starvation conditions in the first five weeks (Figure 8B). The wt cells recovered rapidly when adding the glutamate as the sole N or C source, Δ*gln*B strains showed a growth defect (Figure 8B), compared with the wt strain when adding glutamate, and the cell viability presented a 2.5 log CFU/mL decrease in the following five weeks when adding glutamate as the sole N or C source (Figure 8B). The cell viability was substantially restored in the Δ*gln*B-*C* strain (Figure 8B). The CLSM results showed that, in Δ*gln*B, glutamate did not effectively relieve the inhibition of starvation, as evident by the biomass, which was 5.20 μm^3^/μm^2^ on average (Figure 8C,D); and the average and maximum aggregates thickness were 5.83 and 4.28 μm, respectively (Figure 8C,D). The biomass in Δ*gln*B was two-fold lower than the wt biomass when glutamate was used as the sole N or C source, and Δ*gln*B-*C* effectively restored growth. Based on these results, we inferred that *gln*B was responsible for the recovery from N starvation (Figure 9).

## 4. Discussion

Until recently, the metabolic adaptation of pathogens to utilize host carbon, nitrogen, and energy sources was broadly recognized as a pivotal virulence mechanism [18]. A wide variety of metabolic reprogramming processes occur in pathogens, host cells, and extreme environments to modulate these metabolic adaptations in the pathogen and the survival environment [51]. Despite replicating within endoplasmic reticulum-derived vacuoles, these bacteria do not induce host cell death, pointing to a highly efficient and non-disruptive strategy for resource exploitation [27].

Over the last two decades, mutations in genes related to N metabolism (transporters, enzymes, and regulators) have been found essential for *Brucella* virulence [27]. However, the molecular mechanisms underlying the metabolic adaptation in *B. melitensis* 16M remain largely unknown. In this study, we demonstrated that the OmpR-like regulator FtcR is required for recovery from N starvation in *B. melitensis* 16M, which is supported by the results obtained with complemented strains. ChIP-Seq revealed 363 FtcR-binding sites, which were mapped to GO and KEGG pathways to further investigate the biological functions of these genes. Associated cellular processes are involved in various metabolic and biological pathways. Strikingly, from the top 10 enriched target genes, we identified a novel FtcR-binding site in the promoter region of the N metabolism gene *gln*B, which is responsible for the response to recovery from N starvation, exerting regulatory effect on *gln*B transcription as a strong positive regulator.

FtcR has been reported to regulate complex virulence factors, including FliC, FliF, and FlgE [49], and has been suggested to play an essential role in the virulence of *B. melitensis* 16M [49]. A distinctive structural feature of FtcR is the lack of a canonical phosphorylation site in its receiver domain. Experimental substitution of aspartate with glutamate at the active site can partially mimic phosphorylation, enabling the regulator to bypass its native activation requirement [49]. In addition, a comprehensive genomic inventory of all predicted histidine kinases and response regulators in *B. melitensis* failed to identify a cognate sensor kinase partner for FtcR [49]. One of the additional functions of the FtcR-like regulator was recently reported in a study aimed at identifying new transcription factors involved in exopolysaccharide production, biofilm formation, and nodulation [52]. In this study, we found that FtcR responded to N limitation and was required for recovery from N starvation.

Our results strongly suggested that FtcR functions as a transcriptional regulator. The site of binding typically serves as a marker for the mechanism by which a transcription factor functions; transcription activators generally attach to regions upstream of promoters, while factors that bind at or downstream of the promoter tend to inhibit transcription by obstructing the recruitment of RNA polymerase [53]. FtcR binds upstream from the promoters of flagella, including *fli*C, *fli*F, and *flg*E, consistent with FtcR normally acting as a transcriptional activator for these genes. Genomic sequencing of isolated non-motile *Brucella* species has been used to detect non-functional flagellar genes [54]. In contrast, the examination of genes responsible for flagellar proteins in a motile strain [55] showed that all genes were completely functional, highlighting that certain genes might be expressed in response to environmental selection pressures and carry out specific biological roles [56,57]. One instance of an adaptive function is the triggering of flagellar genes in reaction to changes in the environment or stress conditions [58]. Likewise, the flagellar proteins were upregulated in response to erythritol in the expression of *B. melitensis* genes through microarray assays. However, the exact roles of these flagellar genes in metabolism require further investigation. Notably, in addition to these flagellar genes, FtcR binds upstream of N metabolism-related loci, including *gln*B, *amt*B, and *ntr*Y, suggesting a new role for FtcR in N metabolism.

*Brucella* have developed diverse stress response pathways to improve their assimilation and allocation of limited nutrients [59], such as N [27]. Whether the subcellular dead bacteria material contributes to the survival of adapted *Brucella* remains controversial. Under starvation conditions, the *B. melitensis* 16M growth curve initially declined, contrary to the hypothesis that this is a primary survival strategy involving cannibalism. Although the bacterial survival kinetics were not altered by replacing the culture buffer, indicating a state of persistence, it cannot be completely excluded that during the observed long-term survival in the presence of a low-level balance, bacteria may divide and die, and there may be a low concentration of available C and N sources. Additionally, *Ntr*B and *Ntr*C play critical roles in controlling N metabolism in several bacteria. In contrast to enteric bacteria, it is more likely that the nifR3ntrBC operon represents the *Brucella ntr*BC instead of the *gln*A [27]. Currently, NtrC is unidentified in *Brucella*, but is predicted to bind RpoN and be phosphorylated by NtrB in the cytoplasm (via the uridylated form of PII) at low N concentrations [60]. The *Brucella* NtrYX system senses redox levels through a heme group contained in the PAS domain of the NtrY histidine kinase. As a result of NtrY-phosphorylating NtrX, its cognate response regulators, denitrification genes, are stimulated and expressed under low oxygen tension [61]. An additional gene encoding a lipopolysaccharide core biosynthesis gene is located between *ple*C and *gln*E in all *Brucella* species. In macrophages and mice, a transposition mutant of GlnE is attenuated [32], and high levels of the corresponding protein are found in macrophages at the early stages of infection when *Brucella* is still on its way to the replicative vacuole [62]. GlnD transposon mutants exhibit attenuated virulence in both macrophage and HeLa cell infection models [28]. Interestingly, in *Brucellaceae, glnD* is located upstream of mviN, a gene encoding a putative membrane protein with ten or more transmembrane domains. Although MviN is often classified as a virulence factor, its function extends beyond pathogenesis, indicating a broader biological role. It is essential for *Sinorhizobium meliloti* [63], and is involved in peptidoglycan synthesis [64]. However, deletion of FtcR resulted in a decreased ability to recover from N starvation, indicating its central importance for cells in the process of metabolic adaptation to N starvation. The targets of FtcR signaling in *B. melitensis* 16M are currently unknown, and combined with the ChIP-Seq analysis, we speculated that FtcR regulation via *gln*B might play a role in coordinating metabolic processes with N starvation-related mechanisms. To date, no *Brucella gln*B mutants have been identified. PII signal transduction proteins are one of the oldest and largest families of signaling proteins found in all three domains of life [65]. PII proteins regulate N and C anabolic processes [66]. The PII protein is characterized by a homotrimeric structure featuring three distinct loop regions—B, C, and T—which are responsible for mediating effector molecule binding [66]. Protein interaction modules of PII are characterized by large surface-exposed T-loops and by competitive binding to ADP or ATP; PII proteins determine the energy status of the cell [67]. PII proteins sense the cellular carbon/nitrogen status by detecting ATP levels and the synergistic binding of 2-oxoglutarate. This TCA cycle intermediate serves as a carbon skeleton for inorganic nitrogen assimilation via the GS/GOGAT cycle [68]. FtcR binds upstream from the *glnB* gene that encodes the PII (GlnB) protein, which is only one PII protein in *Brucellaceae*, and it is encoded upstream of *gln*A, a gene for “bona fide” glutamine synthetase [27]. It is conserved in the *Rhizobiaceae*, which has another gene like *gln*B (*gln*K) upstream of the ammonium transporter *amt*B. To investigate the regulatory role of *gln*B in *B. melitensis* 16M, we assessed its transcription by RT-qPCR under varying carbon or nitrogen sources. The results showed that *gln*B was differentially expressed when glutamate was the sole C source or N source, and that the expression levels of *gln*B were similar in the control group in the presence of erythritol as the sole C source or N source. These results are consistent with the growth kinetics results for glutamate and erythritol.

Numerous studies have found that a balanced amino acid proportion is optimal for the in vitro growth of *Brucellae*, whereas little attention has been paid to other organic N sources [26]. Early investigations with *B. abortus* S19 demonstrated that, unlike other amino acids which support minimal growth, glutamate alone can serve as an effective nitrogen source [26]. The dual function of glutamate can be understood through its transformation into α-ketoglutarate, which is subsequently processed via the TCA cycle and gluconeogenesis to generate energy, reducing equivalents, and biosynthetic precursors [27]. Unlike *Enterobacteriaceae*, free-living *Rhizobium* spp. can derive carbon, energy, and nitrogen solely from glutamate, a trait common in this group [69]. We also found similar results in FtcR; that is, although erythritol can be used as the sole C source for the growth of *B. melitensis* 16M, *ftc*R or *gln*B have similar expression levels under erythritol as the sole C source or N source compared with that of the control group. Two non-exclusive hypotheses may explain these results: either *ftc*R*/gln*B activity is condition-specific, or their regulons overlap with environmentally responsive regulators. The latter is plausible, as VjbR—a LuxR-type virulence regulator—is known to be modulated by specific environmental cues [70]. Alternatively, FtcR may require cooperative interactions with other transcriptional cofactors. This is consistent with genomic studies in *E. coli*, where the FNR regulator fails to bind nearly half of its target operons in the absence of specific cofactors [71]. Deletion of *gln*B resulted in slow growth and reduced biomass accumulation in *B. melitensis* 16M during recovery from N starvation.

## 5. Conclusions

We identified a novel target gene for FtcR, *gln*B, encoding a P-II family nitrogen regulator, implying a role for FtcR in the nitrogen or carbon and energy metabolism in *B. melitensis* 16M during long-term nutrient starvation and the ChIP-seq data obtained will facilitate future investigations into the function of FtcR in the global regulation of *B. melitensis* 16M gene expression.

## Figures and Tables

**Table 1:** Primers of qRT-PCR used for this work.

Primer	Sequence	Product Length (bp)
*16S rRNA*-F	ACTAAGGGCGAGGGTTGC	390
*16S rRNA*-R	CACTGGACCATTACTGACGC	
*amtB*-F	AGCCTGCCCGCACTTT	169
*amtB*-R	TCTTGCGCACCATGCC	
*betI*-F	TGCCATCCGCACCATC	182
*betI*-R	GTTTGATCGCCGCGTT	
*glnB*-F	CCAAGGGTTTCGGTC	177
*glnB*-R	AAATCTTGCCGTCGC	
*dksA*-F	CTTCGTGCTCGTGACAGACA	179
*dksA*-R	CGACGCTCATGGCGT	
BME_RS10395-F	CCGAAATGACATGGAAACTT	193
BME_RS10395-R	TCACGCAGATAGGCATCGAA	
BME_RS04690-F	AGAATCAATCGGCTTATG	261
BME_RS04690-R	CTTCCGGCAGCTTGGAGA	
BME_RS13200-F	TCGTCAGAAAGTAATGCAGT	111
BME_RS13200-R	CTCTGGATCTGCGAACGC	
*ntrY*-F	GGCCTTGATCGCTGGTTC	173
*ntrY*-R	ATGAAACCGTTCCGGTCG	
BME_RS08645-F	ATGTCGTGGGAACCTATG	198
BME_RS08645-R	CCAGCGTTTTGCCCTCTT	

**Table 2:** Primers of mutants and complementation used for this work.

Primer	Sequence
*ftcR*-N-F	GCCGAGCCTGAATGTGGAC
*ftcR*-N-R	GACATTCATCCCAGGTGGCCGGCCCCGCCTTCC
*ftcR*-C-F	TCTGGGGTTCGAAATGACCGTATTGATGTTTTCCCGCAAGTC
*ftcR*-C-R	GGGCAGGACAAGCGAGG
*kan*-F	GCCACCTGGGATGAATGTC
*kan*-R	CGGTCATTTCGAACCCCAGA
*glnB*-N-F	AAACCGTCCTTGATGCCG
*glnB*-N-R	GACATTCATCCCAGGTGGCCAGAGAATGGTCTCCGCTCT
*glnB*-C-F	TCTGGGGTTCGAAATGACCGAGCATGTCTCCCAAAAGCG
*glnB*-C-R	TTTCAGATAGGCTTCGGCC
*ftcR*-F-C	AAGCTTGGATGATTGTTGTCGT
*ftcR*-R-C	GAATTCGGTCACTCGATATTGA
*glnB*-F-C	CGTCGACATGAAAAAGATCGAAGCCA
*glnB*-R-C	TGAATTCTTAAATGGCATCCACGC
*p2c2Xamont*	ATATCTAGATTCAGCCGCGGCGGGCT
*p2c2Baval*	ATTGGATCCCCTTCGCGACCGAACCA

**Table 3:** Characterization of the OmpR-type response regulator of *Brucella melitensis* 16M.

Chromosome	Locus Tag	Name	Product Annotation
NC_003317.1	BME_RS02120	*ctrA*	cell cycle two-component system response regulator CtrA
NC_003318.1	BME_RS15125	BME_RS15125	response regulator transcription factor
NC_003318.1	BME_RS14065	BME_RS14065	response regulator transcription factor
NC_003318.1	BME_RS10945	*ftcR*	response regulator transcription factor
NC_003317.1	BME_RS10075	BME_RS10075	response regulator transcription factor
NC_003317.1	BME_RS06730	BME_RS06730	response regulator transcription factor
NC_003317.1	BME_RS06690	BME_RS06690	response regulator transcription factor
NC_003317.1	BME_RS00290	BME_RS00290	response regulator transcription factor
NC_003317.1	BME_RS02475	BME_RS02475	response regulator transcription factor
NC_003318.1	BME_RS10250	BME_RS10250	sigma-54 dependent transcriptional regulator

## Data Availability

The data presented in this study are openly available in NCBI database at https://www.ncbi.nlm.nih.gov/, reference number PRJNA871752.

## Supplementary Materials

The following supporting information can be downloaded at: https://www.mdpi.com/article/10.3390/microorganisms14091898/s1, Figure S1: Assessed the *B. melitensis* 16M viability under starvation conditions and control group; Figure S2: Analysis the survival kinetics of ΔftcR under starvation conditions and control group; Figure S3: Peak distribution analysis of the binding sites of FtcR across the *B. melitensis* 16M genome; Figure S4: GO enrichment network plot of the binding sites of FtcR; Figure S5: KEGG enrichment network plot of the binding sites of FtcR; Figure S6: Bioinformatics analysis of *glnB*.

## Abbreviations

The following abbreviations are used in this manuscript:

PAMPs	pathogen-associated molecular patterns
C	carbon
N	nitrogen
ChIP	chromatin immunoprecipitation
ChIP-Seq	next-generation sequencing
TSA	tryptone soya agar
TSB	tryptone soya broth
BSL-3	Biosafety Level 3
PBS	Phosphate-buffered saline
CLSM	confocal laser scanning microscope
qRT-PCR	quantitative real-time PCR
wt	wild-type
EMSAs	electrophoretic mobility shift assays
