# Supplementary material for: Ability of Brucella melitensis 16M to Control Nitrogen Process: Identification of a Novel P-II Family Nitrogen Regulator
Source: Microorganisms. 2026 Aug 26;14(9):1898. doi: 10.3390/microorganisms14091898 (PMC13609056; doi:10.3390/microorganisms14091898)
Supplement: Supplementary file 1 [file microorganisms-14-01898-s001.zip › microorganisms-4445239-supplementary.pdf]

SUPPORTING INFORMATION

Ability of *Brucella* melitensis 16M to Control Nitrogen Process: Identification of a novel P-II family nitrogen regulator

Yidan Zhang[†,1], Yu Zhang[†,1], Shengnan Song[1], Jing Zhang[1], Zhihua Sun[1,2,3], Xia Zhou[1], Chuangfu Chen[1], Jia Guo[*,1,4] and Hui Zhang[*,1]

[1] State International Joint Research Center for Animal Health Breeding, College of Animal Science and Technology, Shihezi University, Shihezi 832003, China

[2] Huyanghe City Haoyuanxin Animal Husbandry Development Co., Ltd., Huyanghe 834034, China

[3] Xinjiang Shawan Tianrun Co., Ltd., Shawan 832100, China

[4] Xinjiang Aheqi Agriculture and Animal Husbandry Development Co., Ltd., Aheqi 843500, China

[*] Correspondence: zhguojia@stu.shzu.edu.cn (J.G.); shz_zhang@126.com (H.Z.)

[†] These authors contributed equally to this work

Supplementary Figures

Figure S1 Assessed the *B. melitensis* 16M viability under starvation conditions and control group. (A) Live confocal imaging of *B. melitensis* 16M cells labeled using Syto9 and PI fluorescent probes. (B) Survival of bacteria under starvation conditions and control group. Error bars represent standard error ($n \geq 3$). $^{**}p \leq 0.01$, unpaired Student's *t* test.

Figure S2 Analysis the survival kinetics of Δ*ftcR* under starvation conditions and control group. (A) Live confocal imaging of stacks of *B. melitensis* 16M-GFP grown for five weeks in starvation or in adding glutamate conditions. Scale bars = 20 μm. (B, C) Confocal images were subjected to quantitative analysis using the Comstat2 program to determine the biomass (B) and the average and maximum thickness (C) in starvation conditions or in adding glutamate conditions. Error bars represent standard error ($n \geq 3$). $^{**}p \leq 0.01$, unpaired Student's *t* test.

Figure S3 Peak distribution analysis of the binding sites of FtcR across the *B. melitensis* 16M genome. (A) Peak length distribution analysis of the binding sites of FtcR across the *B. melitensis* 16M genome. (B) Histogram and fitting line of distance frequencies from peak summit to the nearest start codon for the 363 *bona fide* ChIP-Seq peaks localized at intergenic (blue) or coding regions (red). The dotted line indicates the relative position of the start codon.

Figure S4 GO enrichment network plot of the binding sites of FtcR. A greater rich factor indicates a greater degree of pathway enrichment.

Figure S5 KEGG enrichment network plot of the binding sites of FtcR. A greater rich factor indicates a greater degree of pathway enrichment.

Figure S6 Bioinformatics analysis of *glnB.* (A) The *glnB* chromosome location information. (B) Prediction of the functional domain of *glnB* protein in *B. melitensis* 16M. (C) Construction of a protein-protein correlation network according to the prediction of potential targets using protein interaction network analysis.

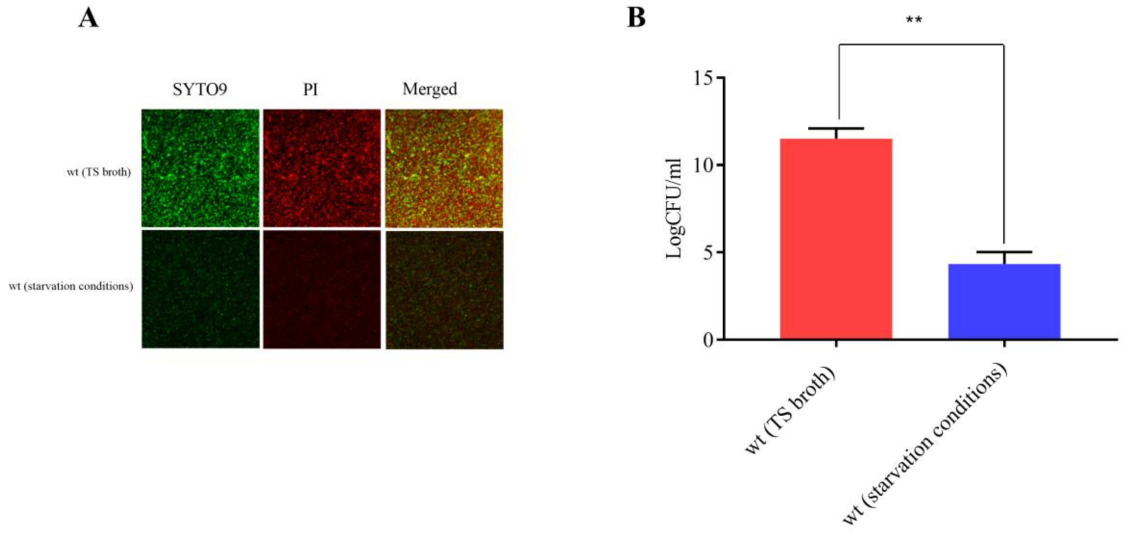

Figure S1 Assessed the *B. melitensis* 16M viability under starvation conditions and control group. (A) Live confocal imaging of *B. melitensis* 16M cells labeled using Syto9 and PI fluorescent probes. (B) Survival of bacteria under starvation conditions and control group. Error bars represent standard error (n ≥ 3). $^{**}p \leq 0.01$, unpaired Student's *t* test.

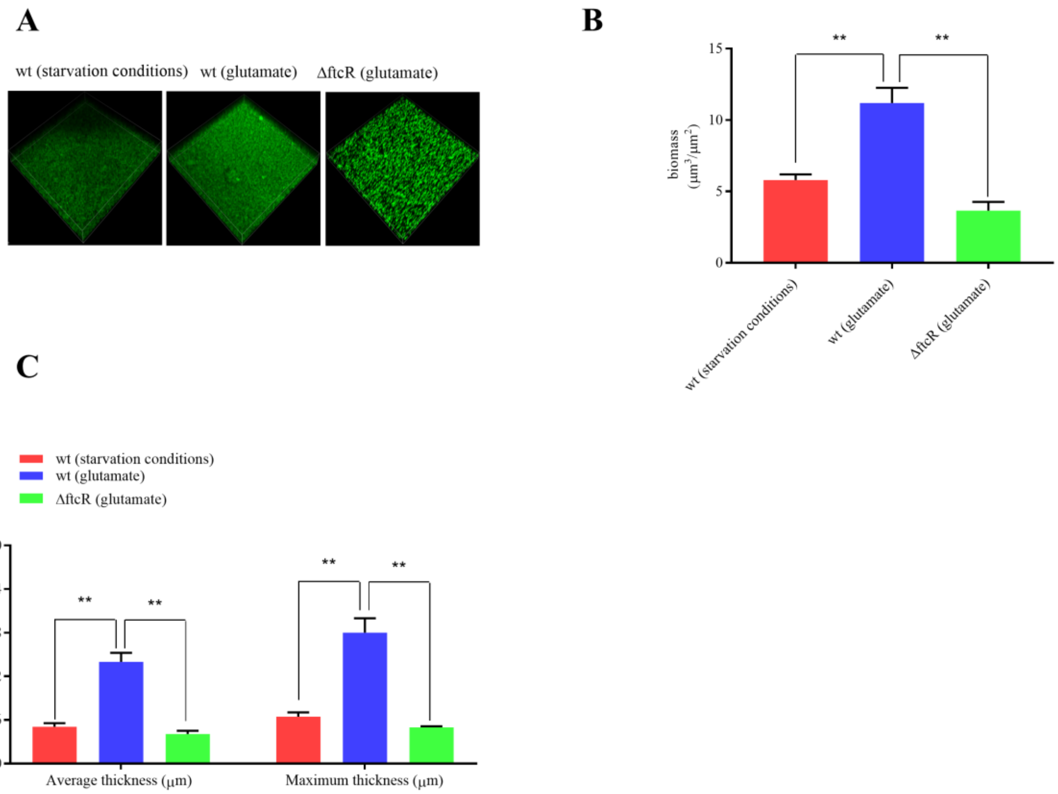

Figure S2 Analysis the survival kinetics of Δ*ftcR* under starvation conditions and control group.(A) Live confocal imaging of stacks of *B. melitensis* 16M-GFP grown for five weeks in starvation or in adding glutamate conditions. Scale bars = 20 μm. (B, C) Confocal images were subjected to quantitative analysis using the Comstat2 program to determine the biomass (B) and the average and maximum thickness (C) in starvation conditions or in adding glutamate conditions. Error bars represent standard error ($n \geq 3$). $^{**}p \leq 0.01$, unpaired Student's *t* test.

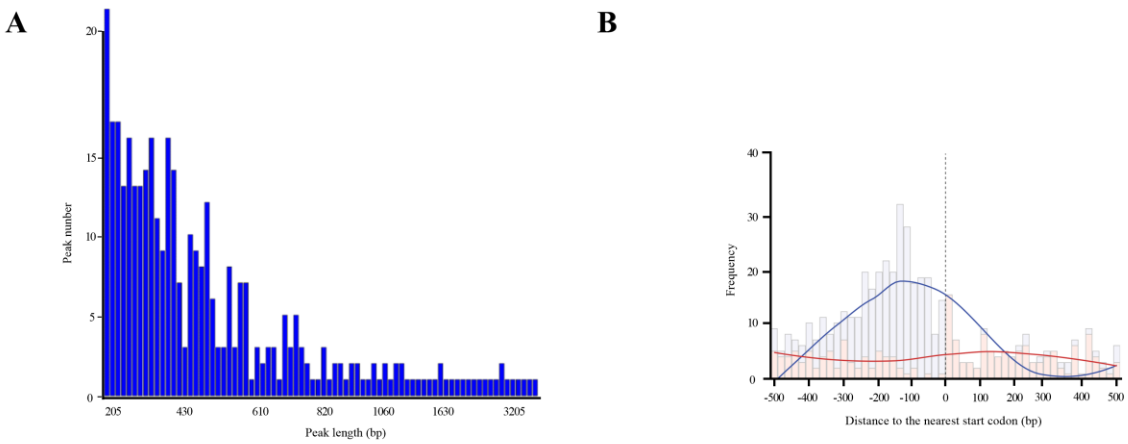

Figure S3 Peak distribution analysis of the binding sites of FtcR across the *B. melitensis* 16M genome. (A) Peak length distribution analysis of the binding sites of FtcR across the *B. melitensis* 16M genome. (B) Histogram and fitting line of distance frequencies from peak summit to the nearest start codon for the 363 *bona fide* ChIP-Seq peaks localized at intergenic (blue) or coding regions (red). The dotted line indicates the relative position of the start codon.

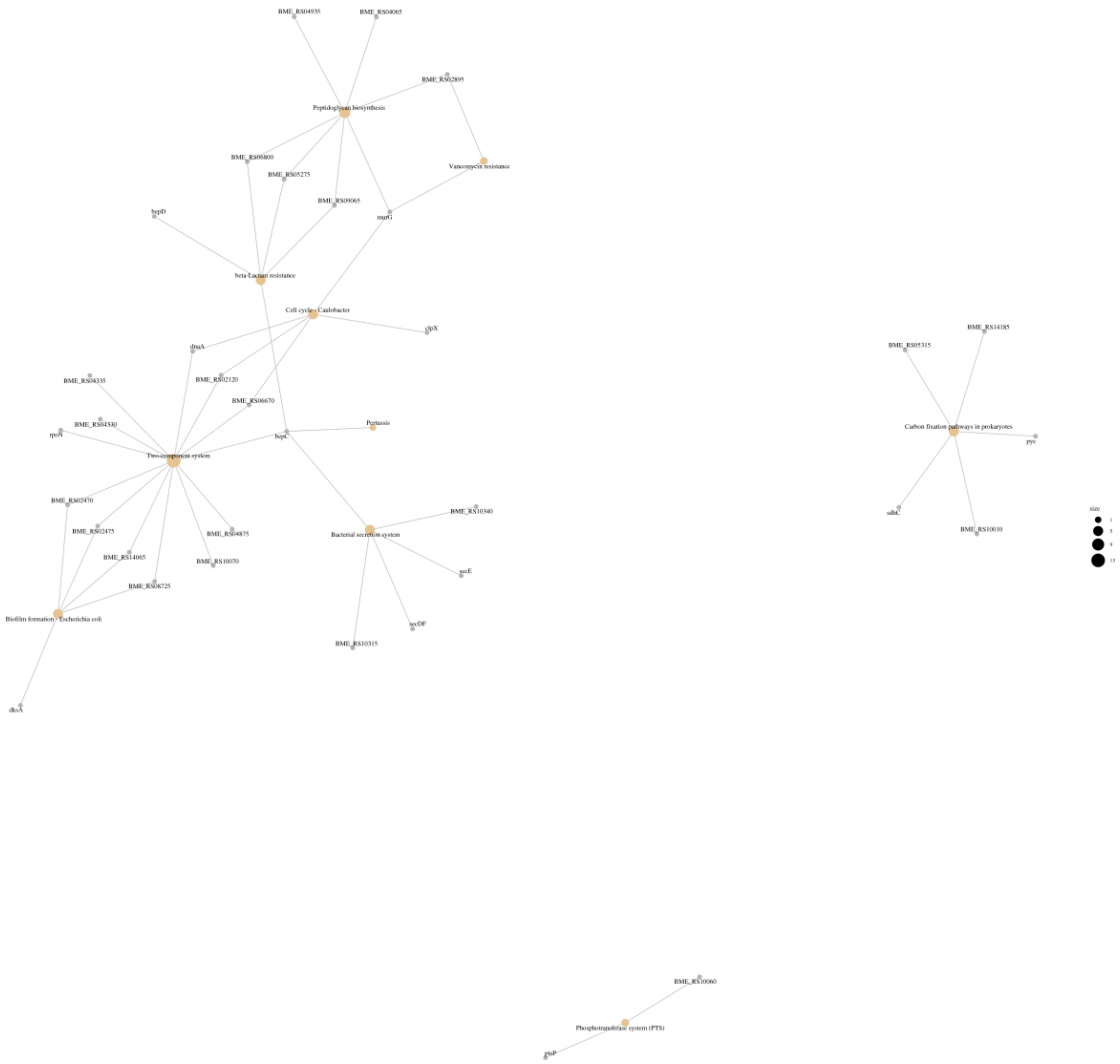

Figure S4 GO enrichment network plot of the binding sites of FtcR. A greater rich factor indicates a greater degree of pathway enrichment.

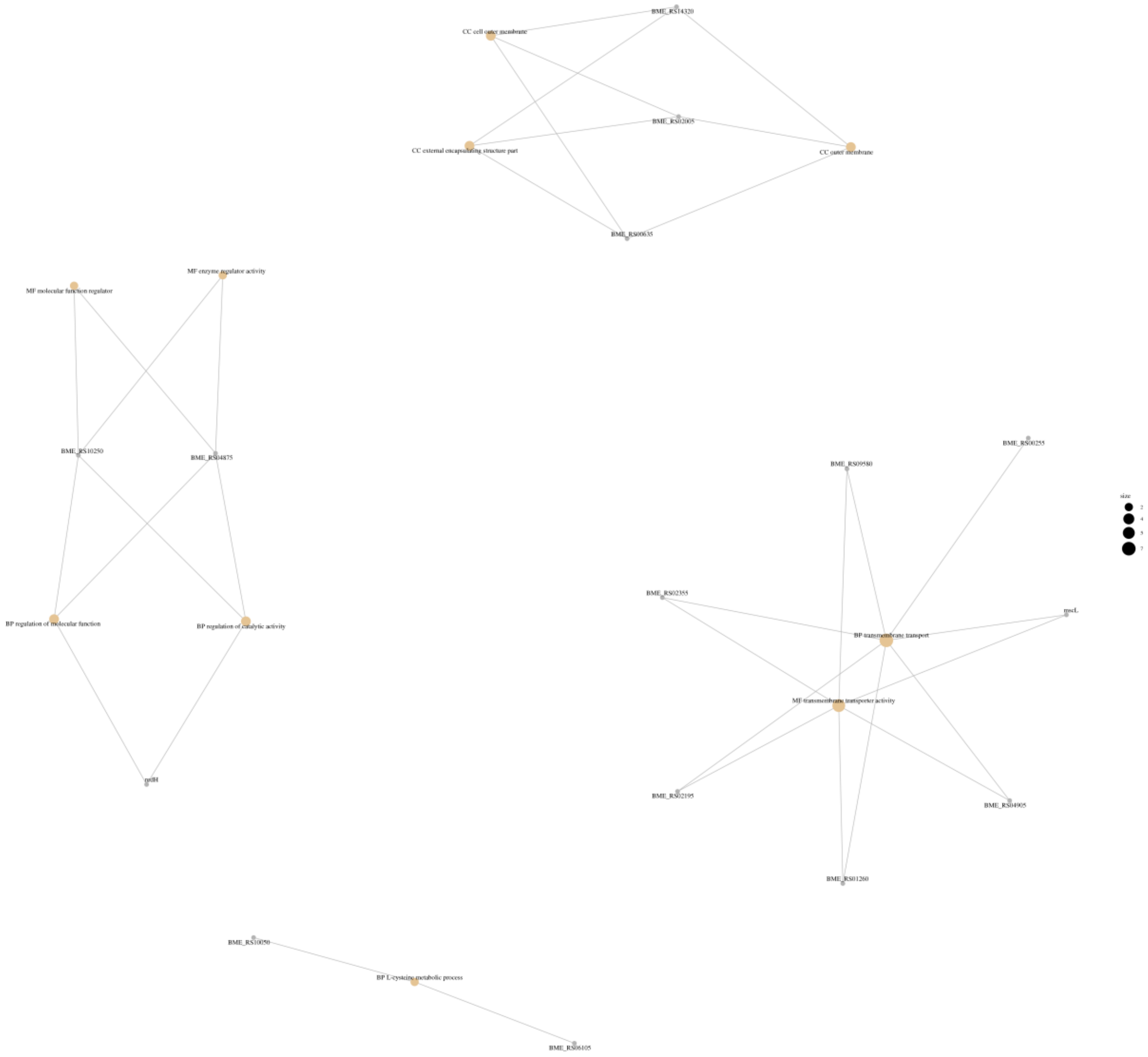

Figure S5 KEGG enrichment network plot of the binding sites of FtcR. A greater rich factor indicates a greater degree of pathway enrichment.

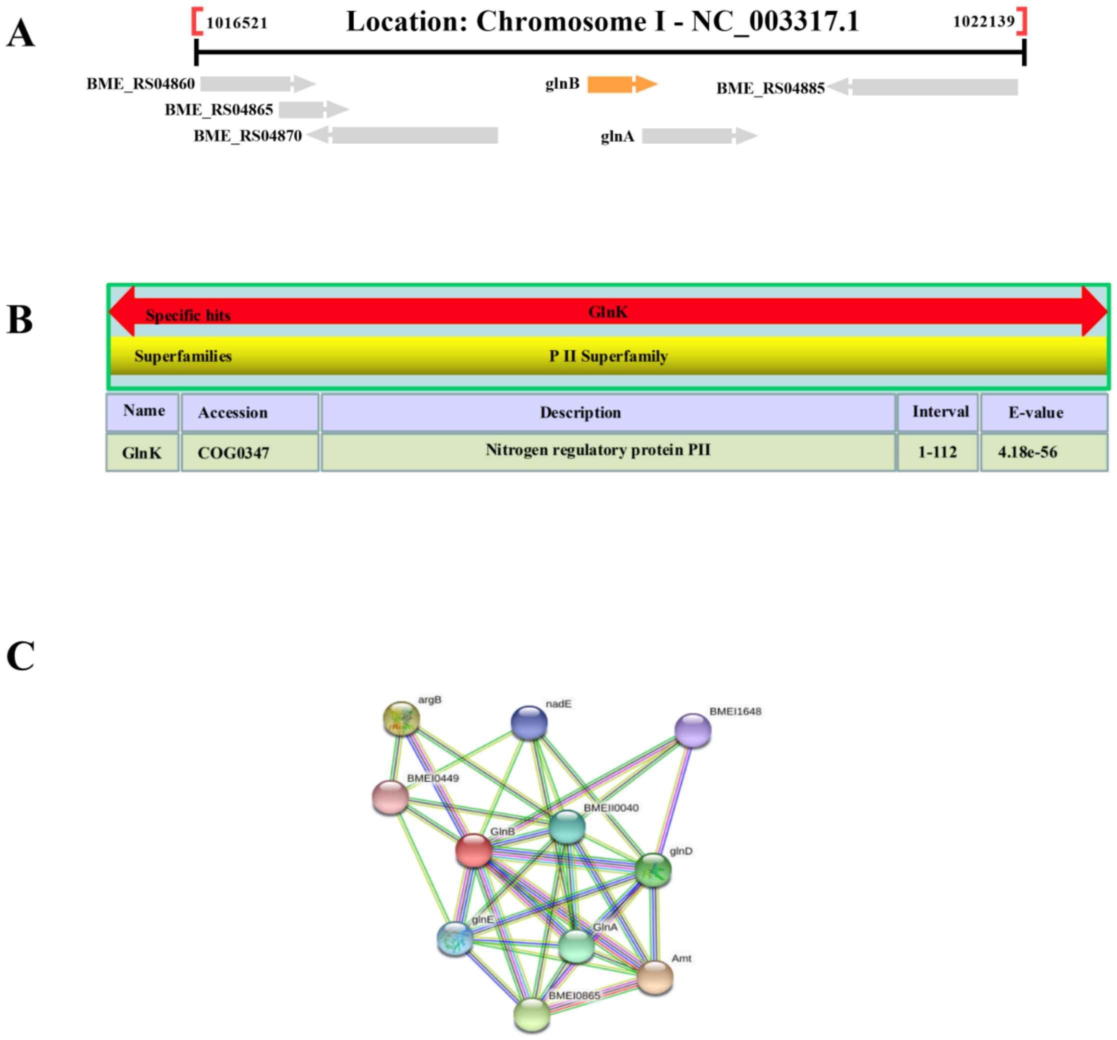

Figure S6 Bioinformatics analysis of *glnB*. (A) The *glnB* chromosome location information. (B) Prediction of the functional domain of *glnB* protein in *B. melitensis* 16M. (C) Construction of a protein-protein correlation network according to the prediction of potential targets using protein interaction network analysis.
